# Supplementary figures and images for: Direct and indirect effects of age on dengue severity: The mediating role of secondary infection
Source: PLoS Negl Trop Dis. 2023 Aug 9;17(8):e0011537. doi: 10.1371/journal.pntd.0011537 (PMC10441797; doi:10.1371/journal.pntd.0011537)

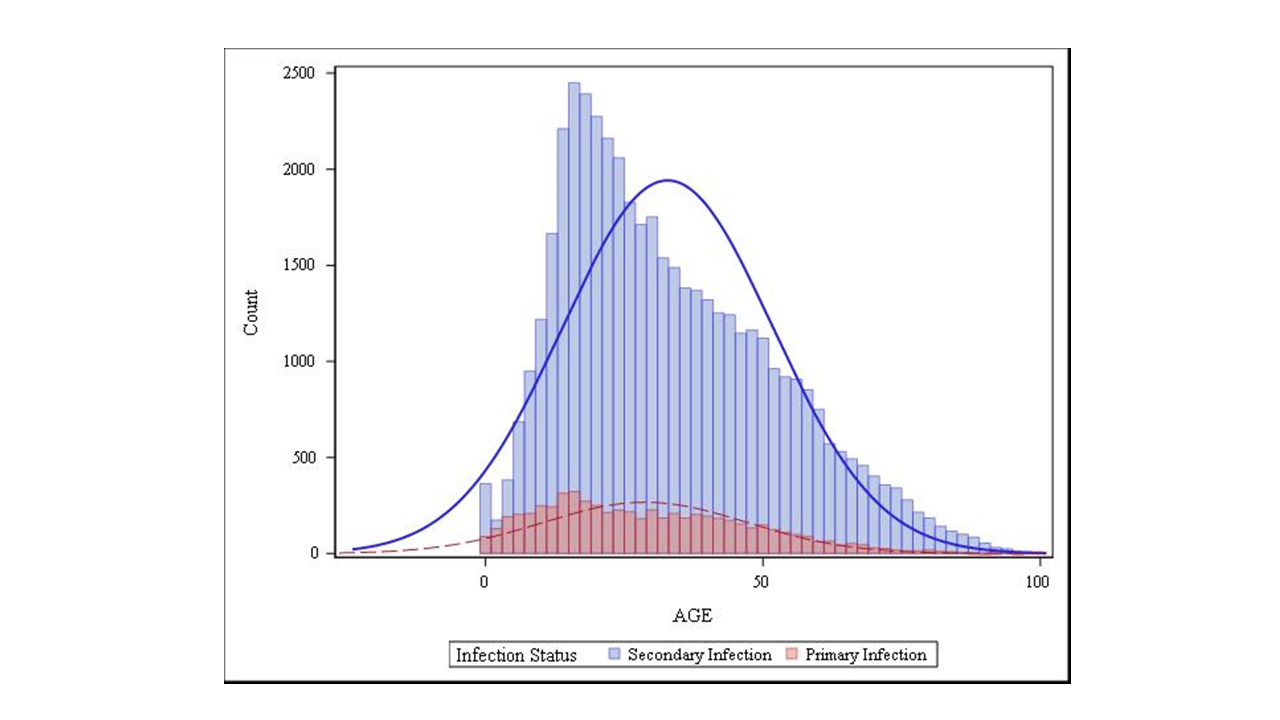

Supplement: S1 Fig — (TIF) [file pntd.0011537.s001.tif]

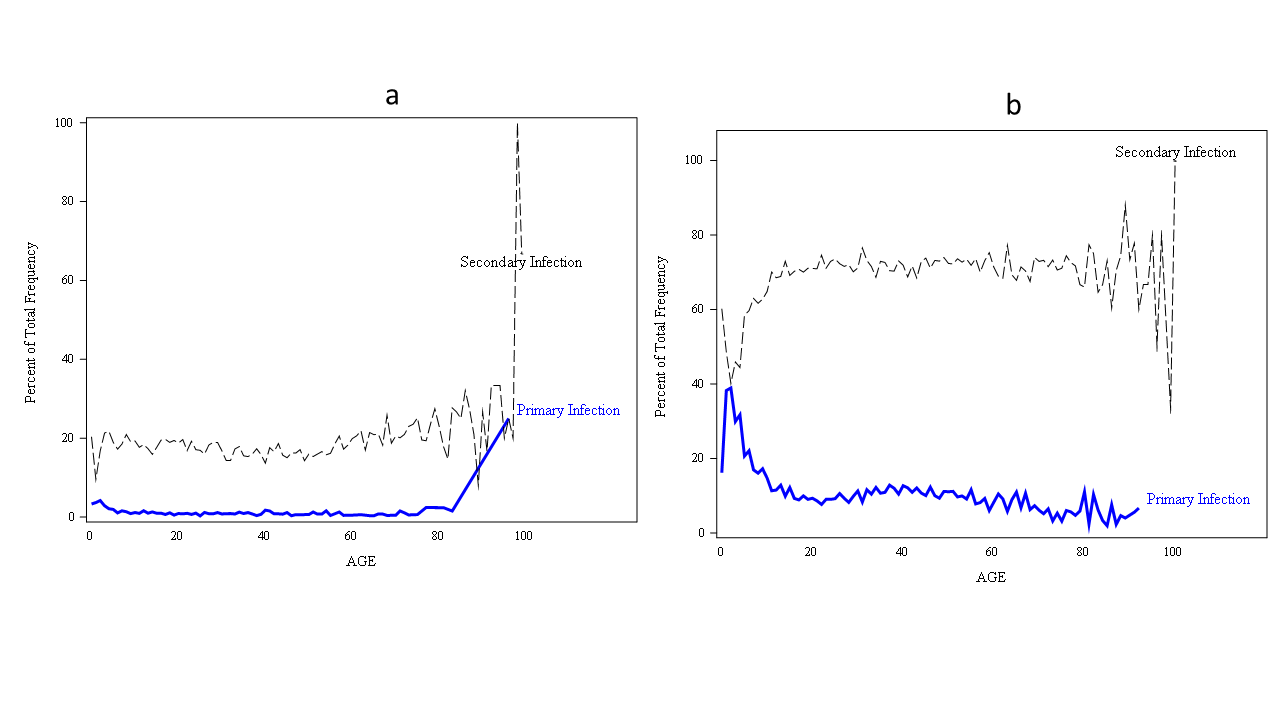

Supplement: S2 Fig — a: Frequency polygon of Age pattern for severe dengue. b: Frequency polygon of Age pattern for non-severe dengue. (TIF) [file pntd.0011537.s002.tif]

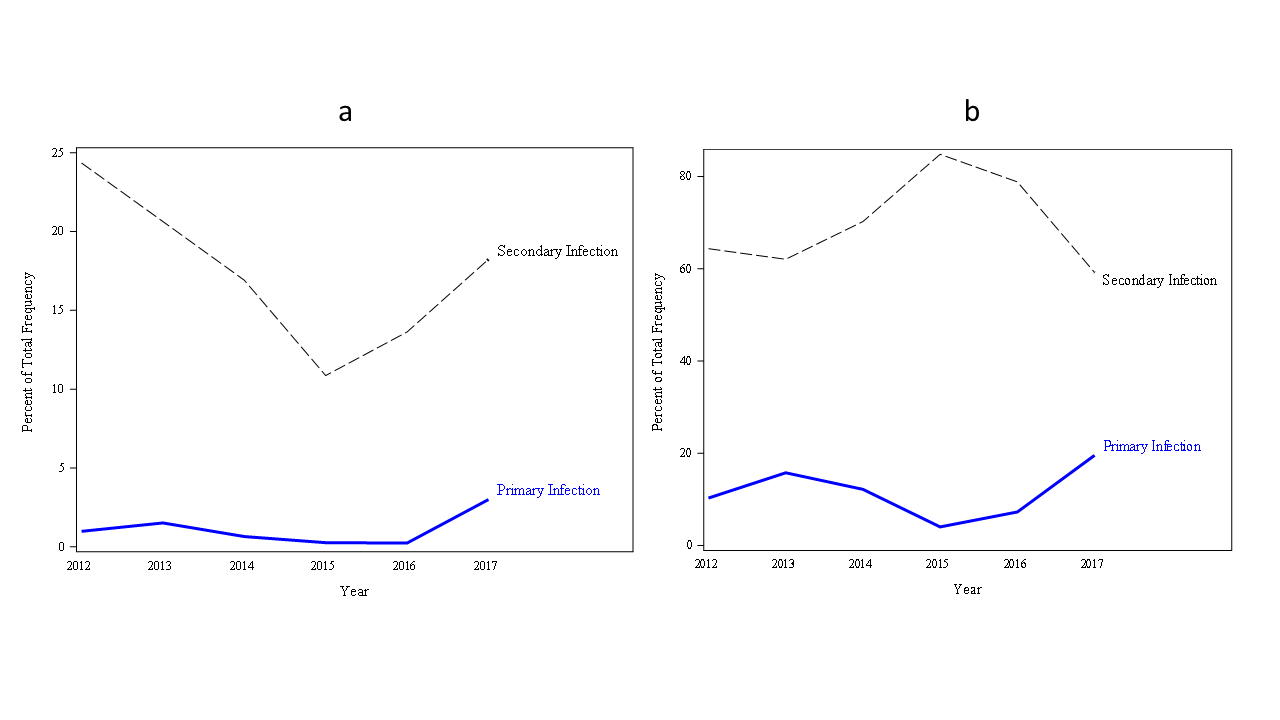

Supplement: S3 Fig — a: Trend of infection status from 2012 to 2017 for severe dengue. b: Trend of infection status from 2012 to 2017 for non-severe dengue. (TIF) [file pntd.0011537.s003.tif]

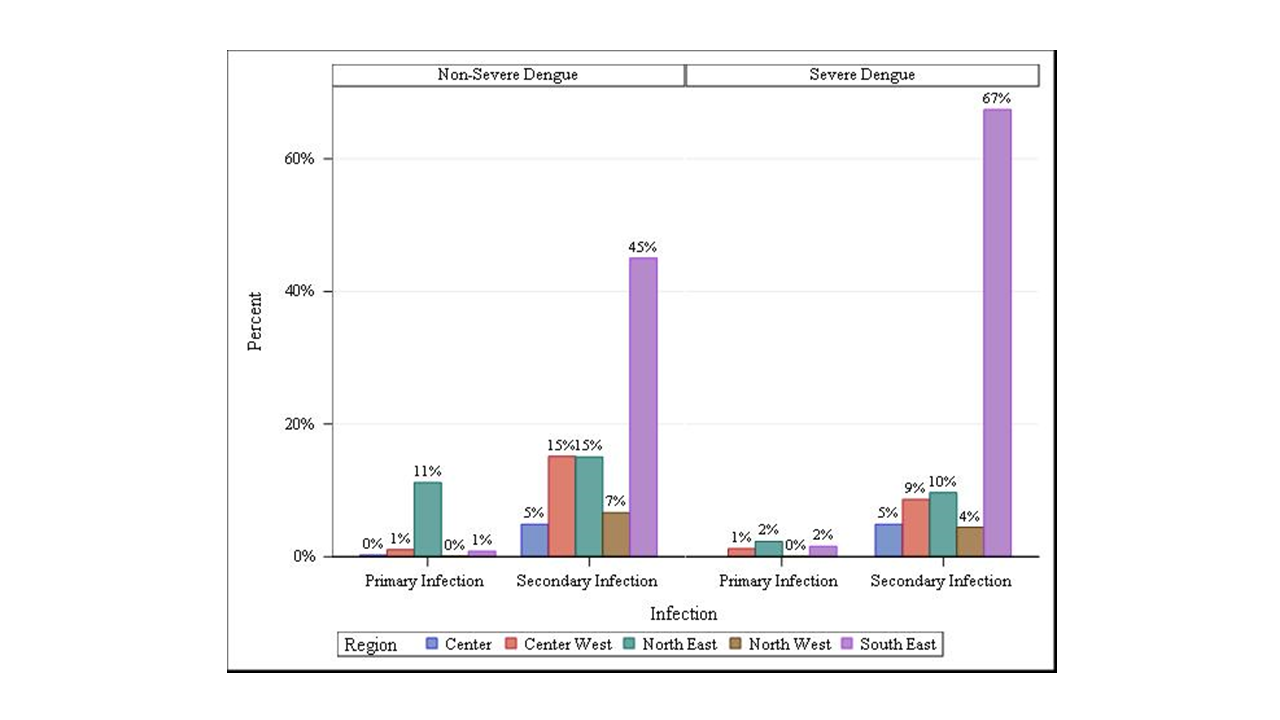

Supplement: S4 Fig — (TIF) [file pntd.0011537.s004.tif]

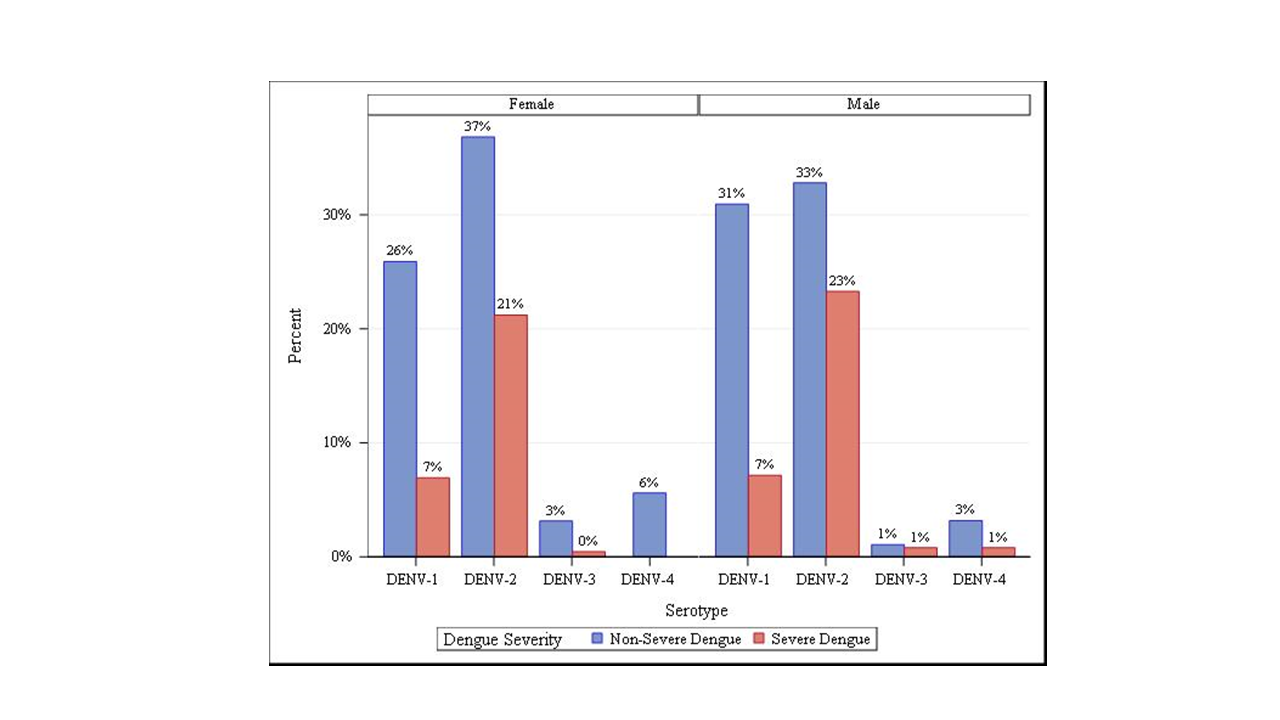

Supplement: S5 Fig — (TIF) [file pntd.0011537.s005.tif]

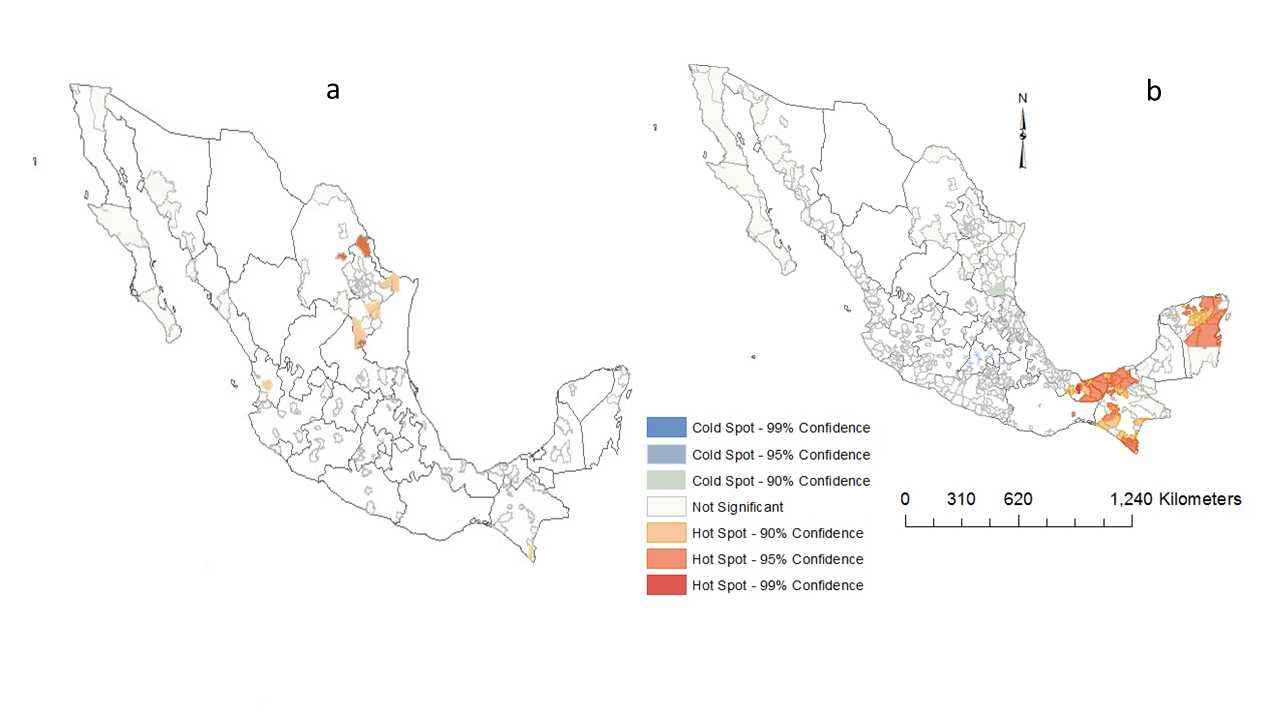

Supplement: S6 Fig — a: Severe Dengue Hotspot among individuals with primary infection. b: Severe Dengue Hotspot among individuals with secondary infection. Map: https://www.diva-gis.org/gdata. (TIF) [file pntd.0011537.s006.tif]
