# Supplementary material for: Direct and indirect effects of age on dengue severity: The mediating role of secondary infection
Source: PLoS Negl Trop Dis. 2023 Aug 9;17(8):e0011537. doi: 10.1371/journal.pntd.0011537 (PMC10441797; doi:10.1371/journal.pntd.0011537)
Supplement: S1 Table — (DOCX) [file pntd.0011537.s007.docx]

S1_Table: Demographic characteristics by infection classification

^#^ Chi-square performed for sex differences, p = 0.9447

^+^ P-value of T- test <= 0.0001

| Parameter | Infection Classification, n (%) | | | |
| --- | --- | --- | --- | --- |
|  |  | Primary | Secondary | All |
| ^#^Sex | Male | 2455 (39.96%) | 18433 (40.01%) | 20888 (40.01%) |
|  | Female | 3689 (60.04%) | 27635 (59.99%) | 31324 (59.99%) |
| ^+^ Region | Center | 96 (1.56%) | 2543 (5.51%) | 2635 (5.05%) |
|  | Center- West | 561 (9.13%) | 7260 (15.76%) | 7821 (14.98%) |
|  | North - East | 4949 (80.55%) | 7324 (15.90%) | 12273 (23.51%) |
|  | North - West | 38 (0.62%) | 3234 (7.02%) | 3272 (6.27%) |
|  | South - East | 500 (8.14%) | 25711 (55.81%) | 26211 (50.20%) |
| ^+^Age in years (SD) |  | 28 (±18.38) | 32 (±18.95) | 32 (±18.92) |
| Total (n) |  | 6144 (11.77%) | 46068 (88.23%) | 52212 (100%) |
