# Supplementary material for: Direct and indirect effects of age on dengue severity: The mediating role of secondary infection
Source: PLoS Negl Trop Dis. 2023 Aug 9;17(8):e0011537. doi: 10.1371/journal.pntd.0011537 (PMC10441797; doi:10.1371/journal.pntd.0011537)
Supplement: S2 Table — (DOCX) [file pntd.0011537.s008.docx]

S2 Table: Relationship between the region and serotype distribution.

| Parameter |  | Region | | | | |
| --- | --- | --- | --- | --- | --- | --- |
|  |  | Center | Center west | Northeast | Northwest | Southeast |
| ^+^Serotype | DENV-1 | 1913 (10.82%) | 8178 (46.26%) | 1992 (11.27%) | 970 (5.49%) | 4627 (26.17%) |
|  | DENV-2 | 763 (8.08%) | 2283 (24.19%) | 1872 (19.83%) | 578 (6.12%) | 3943 (41.77%) |
|  | DENV-3 | 63 (12.09%) | 7 (1.34%) | 220 (42.23%) | 3 (0.58%) | 228 (43.76%) |
|  | DENV-4 | 9 (1.85%) | 32 (6.60%) | 116 (23.92%) | 0 (0.00%) | 328 (67.63%) |

^+^ P-value of significance test < 0.0001
