# Supplementary material for: Direct and indirect effects of age on dengue severity: The mediating role of secondary infection
Source: PLoS Negl Trop Dis. 2023 Aug 9;17(8):e0011537. doi: 10.1371/journal.pntd.0011537 (PMC10441797; doi:10.1371/journal.pntd.0011537)
Supplement: S3 Table — (DOCX) [file pntd.0011537.s009.docx]

S3 Table: Generalized additive model of the effect of age on infectivity status showing covariate results.

| Variable | OR | 95% Confidence Interval |
| --- | --- | --- |
| Sex |  |  |
| Male vs Female | 0.91 | 0.86, 0.97 |
| Region |  |  |
| Center West vs Center | 0.51 | 0.41, 0.63 |
| Northeast vs Center | 0.05 | 0.04, 0.06 |
| Northwest vs Center | 3.10 | 2.30, 4.51 |
| Southeast vs Center | 2.19 | 1.76, 2.73 |
