# Supplementary material for: Direct and indirect effects of age on dengue severity: The mediating role of secondary infection
Source: PLoS Negl Trop Dis. 2023 Aug 9;17(8):e0011537. doi: 10.1371/journal.pntd.0011537 (PMC10441797; doi:10.1371/journal.pntd.0011537)
Supplement: S4 Table — (DOCX) [file pntd.0011537.s010.docx]

S4 Table: Generalized additive model of the effect of age on dengue severity showing covariate results.

| Variable | OR | 95% Confidence Interval |
| --- | --- | --- |
| Sex |  |  |
| Male vs Female | 1.27 | 1.25, 1.30 |
| Region |  |  |
| Center West vs Center | 0.69 | 0.66, 0.72 |
| Northeast vs Center | 0.51 | 0.49, 0.53 |
| Northwest vs Center | 0.67 | 0.64, 0.69 |
| Southeast vs Center | 1.84 | 1.77, 1.92 |
