# Supplementary material for: Direct and indirect effects of age on dengue severity: The mediating role of secondary infection
Source: PLoS Negl Trop Dis. 2023 Aug 9;17(8):e0011537. doi: 10.1371/journal.pntd.0011537 (PMC10441797; doi:10.1371/journal.pntd.0011537)
Supplement: S5 Table — (DOCX) [file pntd.0011537.s011.docx]

S5 Table: Generalized additive model of the effect of age and infectivity status on dengue severity showing covariate results.

| Variable | OR | 95% Confidence Interval |
| --- | --- | --- |
| Sex |  |  |
| Male vs Female | 1.40 | 1.35, 1.46 |
| Region |  |  |
| Center West vs Center | 0.64 | 0.57, 0.72 |
| Northeast vs Center | 0.57 | 0.51, 0.64 |
| Northwest vs Center | 0.69 | 0.60, 0.79 |
| Southeast vs Center | 1.56 | 1.41, 1.72 |
| Infectivity Status |  |  |
| Secondary vs Primary | 1.71 | 1.55, 1.89 |
