# Supplementary material for: Direct and indirect effects of age on dengue severity: The mediating role of secondary infection
Source: PLoS Negl Trop Dis. 2023 Aug 9;17(8):e0011537. doi: 10.1371/journal.pntd.0011537 (PMC10441797; doi:10.1371/journal.pntd.0011537)
Supplement: Attachment — Submitted filename: PNTD-D-22-01621_review.pdf [file pntd.0011537.s012.pdf]

PLOS Neglected Tropical Diseases

Direct and indirect effects of age on dengue severity: the mediating role of secondary infection

--Manuscript Draft--

**Manuscript Number:**	PNTD-D-22-01621
**Full Title:**	Direct and indirect effects of age on dengue severity: the mediating role of secondary infection
**Short Title:**	Mediating role of secondary infection on severe dengue
**Article Type:**	Research Article
**Keywords:**	dengue fever; severe dengue; secondary infection; mediation; serotype; direct and indirect effect; Mexico
**Abstract:**	Severe dengue occurrence has been attributed to increasing age and different dengue virus (DENV) serotypes that cause secondary infections and immune-enhancing phenomena. Therefore, we examined if the effect of age on dengue severity was mediated by infectivity status while controlling for sex and DENV-specific serotypes. Further, we assessed the spatial clustering of dengue severity for individuals with primary and secondary infection across Mexican municipalities. Health data from 2012 to 2017 was retrieved from Mexico's Ministry of Health. A mediation analysis was performed using multiple logistic regression models based on a directed acyclic graph. The models were explored for the direct effect of age on dengue severity and its indirect impact through secondary infection. In addition, severe dengue hotspots were determined through spatial analysis. While people between 21-40 years [0.82, (CI: 0.77, 0.88)] and those between 41-60 years [0.82, (CI: 0.76, 0.89)] had lower odds of severe dengue, those who were 61 years and older [1.19, (CI: 1.05, 1.35)] had higher odds of severe dengue when compared with those less than 21 years old. The effect of age on dengue severity was no longer significant after introducing infectivity status into the logistic regression model, indicating age effect was largely mediated by secondary infection. Clustering of severe dengue among individuals with primary infection in the Northeastern region may point to the high prevalence of DENV-3 in the region. Public health efforts may prevent secondary infection among infants and the aged. In addition, there should be a further investigation into the effect of DENV-3 in individuals with primary disease.
**Additional Information:**	
**Question**	**Response**
**Government Employee**<br><br>Are you or any of the contributing authors an employee of the United States government?<br><br>Manuscripts authored by one or more US Government employees are not copyrighted, but are licensed under a CC0 Public Domain Dedication, which allows unlimited distribution and reuse of the article for any lawful purpose. This is a legal requirement for US Government employees.	No - No authors are employees of the U.S. government.

Powered by Editorial Manager® and ProduXion Manager® from Aries Systems Corporation

This will be typeset if the manuscript is accepted for publication.	
**Financial Disclosure**<br><br>Enter a financial disclosure statement that describes the sources of funding for the work included in this submission and the role the funder(s) played. This includes grants and any commercial funding of the work or authors.<br><br>This statement will be typeset if the manuscript is accepted for publication.<br><br>Review the submission guidelines and the instructions link below for detailed requirements and guidance.	There was no funding for this study. However, U.H. was supported by Rutgers Global Health Institute.
**Competing Interests**<br><br>On behalf of all authors, disclose any competing interests that could be perceived to bias this work.<br><br>This statement will be typeset if the manuscript is accepted for publication.<br><br>Review the instructions link below and PLOS NTDs' competing interests policy to determine what information must be disclosed at submission.	The authors declare no conflict of interest.
**Data Availability**<br><br>Provide a **Data Availability Statement** in the box below. This statement should detail where the data used in this submission can be accessed. This statement will be typeset if the manuscript is accepted for publication.<br><br>Before publication, authors are required to make all data underlying their findings fully available, without restriction. Review our PLOS Data Policy page for detailed	Data may be found in the Zenodo repository (Link will be provided after acceptance)

information on this policy. Instructions for writing your Data Availability statement can be accessed via the Instructions link below.	

Powered by Editorial Manager® and ProduXion Manager® from Aries Systems Corporation

Manuscript Click here to access/download;Manuscript;MS_mediation_analysis.docx

Direct and indirect effects of age on dengue severity: the mediating role of secondary infection

Esther Annan[1], Jesús Treviño[2], Bingxin Zhao[3], Alfonso J. Rodriguez-Morales[4,5,6], Ubydul Haque[7,8].

[1]Center for Health and Wellbeing, School of Public and International Affairs, Princeton University, Princeton, NJ, United States

[2]Department of Urban Affairs at the School of Architecture, Universidad Autónoma de Nuevo León, San Nicolás de los Garza, 66455, Nuevo Léon, México

[3]Department of Statistics and Data Science, The Wharton School, University of Pennsylvania, Philadelphia, PA 19104, United States

[4]Grupo de Investigacion Biomedicina, Faculty of Medicine, Fundacion Universitaria Autónoma de las Americas, Pereira, Risaralda, Colombia.

[5]Faculty of Medicine, Institución Universitaria Visión de las Americas, Pereira, Risaralda, Colombia.

[6]Clinical Epidemiology and Biostatistics, Universidad Cientifica del Sur, Lima, Peru.

[7]Rutgers Global Health Institute, New Brunswick, NJ

[8]Department of Biostatistics and Epidemiology, School of Public Health, Rutgers University, Piscataway, NJ, USA

Keywords: dengue fever; severe dengue; secondary infection; mediation; serotype; direct and indirect effect; Mexico

*Corresponding Author
E-mail: esther.annan@princeton.edu

Abstract

Severe dengue occurrence has been attributed to increasing age and different DENV serotypes that cause secondary infections and immune-enhancing phenomena. Therefore, we examined if the effect of age on dengue severity was mediated by infectivity status while controlling for sex and DENV-specific serotypes. Further, we assessed the spatial clustering of dengue severity for individuals with primary and secondary infection across Mexican municipalities. Health data from 2012 to 2017 was retrieved from Mexico's Ministry of Health. A mediation analysis was performed using multiple logistic regression models based on a directed acyclic graph. The models were explored for the direct effect of age on dengue severity and its indirect impact through secondary infection. In addition, severe dengue hotspots were determined through spatial analysis. While people between 21-40 years [0.82, (CI: 0.77, 0.88)] and those between 41-60 years [0.82, (CI: 0.76, 0.89)] had lower odds of severe dengue, those who were 61 years and older [1.19, (CI: 1.05, 1.35)] had higher odds of severe dengue when compared with those less than 21 years old. The effect of age on dengue severity was no longer significant after introducing infectivity status into the logistic regression model, indicating age effect was largely mediated by secondary infection. Clustering of severe dengue among individuals with primary infection in the Northeastern region may point to the high prevalence of DENV-3 in the region. Public health efforts may prevent secondary infection among infants and the aged. In addition, there should be a further investigation into the effect of DENV-3 in individuals with primary disease.

Synopsis

Age has been previously found to be associated with severe dengue. However, it was unclear whether this effect was purely due to aging or the predisposition of older individuals to be exposed to secondary infection. Since secondary infection with a dengue serotype other than the infecting serotype during the first infection increases the risk of severe dengue, we explored how much this effect contributed to the effect of age on severe dengue. We performed a mediation analysis and assessed the spatial distribution pattern of primary and secondary infection across Mexico to determine regions vulnerable to severe dengue occurrence. The youngest and oldest population groups had relatively higher odds of severe dengue. However, the effect of age on severe dengue was largely mediated by secondary infection. The spatial analysis also revealed aggregation of severe dengue cases among individuals with primary infection in the Northeastern region, which may be associated with the high prevalence of DENV-3 in the region. Focusing on the prevention of secondary infection among the elderly and infants may help to avert the number of severe dengue cases.

Introduction

Dengue fever has in recent years expanded in geographic distribution, causing endemic diseases with seasonal fluctuations across the globe [1-4]. In Mexico, the force of dengue infection between 2008 and 2014 was about 7.1% (5.1%–9.8%), indicating a high endemic transmission [5]. Infection with dengue presents on a spectrum of mild to severe, with economic impact primarily attributable to indirect costs from loss of productivity [6]. The seasonal transmission of dengue may be explained by environmental factors [7], viral evolution, and population-specific immunity against different DENV serotypes [8]. Individual-level predictors of disease severity have been found to include age, comorbidities, sex, and infection with multiple serotypes [9-14].

After a first epidemic, subsequent epidemics result in fewer cases due to herd immunity, and younger individuals become progressively susceptible as the immuned-population ages [15]. As epidemics continue over time, the age-specific seroprevalence is expected to change, with small outbreaks involving ~~the~~ younger ~~generation~~ [15]. The risk of having a classical dengue disease, which traditionally reflects less severe symptoms, has been found to increase with age after a person experiences primary infection [16].

Primary dengue infection is characterized by high titers of immunoglobulin M (IgM) in 3-5 days and immunoglobulin G (IgG) antibodies from the sixth to the tenth day after symptom onset [17]. While the IgM disappears in 2-3 months, IgG persists in the body for life, providing lifelong immunity against the infecting serotype, but not the other three dengue serotypes [17]. The same serotype as the first infection, or any of the other three ~~serotypes~~ may cause secondary dengue infections. When a person ~~gets~~ re-infected with a different or unencountered DENV serotype, this usually leads to classical dengue fever (DF) or dengue without warning signs [17]. Of the people with a secondary infection, 2% - 3% progress to dengue hemorrhagic fever (DHF), which may further result in dengue shock syndrome (DSS) and death [17]. DHF is characterized by hemorrhagic signs like thrombocytopenia, petechiae and epistaxis, while DSS occurs due to leakage of intravascular fluids and proteins into perivascular spaces [18]. Antibody-dependent enhancement (ADE) occurs because of secondary infection with a heterologous DF serotype and is

associated with severer infections. However, not all severe infections are a result of a secondary infection [17].

An individual's exposure to a secondary infection increases with age, and the outcome of dengue serotype 2 (DENV-2) secondary infection has been found to predict the risk of severe clinical presentation of DF [19]. The association between secondary serologic response and severe dengue has been established in the previous literature [20], and prior studies have explored the influence of age on clinical dengue presentation. Age has been found to be associated with an increased risk of clinical dengue [1]. However, to our knowledge, the mediation effect of secondary infection in the causal pathway between age and severe dengue has not been studied. Hence, our study aimed to quantify age's direct and indirect effect on dengue severity by mediating secondary infection (Figure 1). We then further characterized the effect of secondary infection on dengue severity by exploring the rate of secondary infection across time and space in Mexico.

Methods

Data Collection and Preprocessing

Data used for the analysis was acquired from the Ministry of Health, Mexico. Data for all variables of interest were compiled from 2012 to 2017, and a cross-sectional study was performed. Hence, the analysis was restricted to observations from these six years. The Ministry of Health data reports non-identifiable clinical findings from notifiable health units in 2,469 Mexican municipalities. Therefore, this study was approved as an exempt category by the North Texas Regional Institutional Review Board and the Universidad Autónoma De Nuevo León.

Definition of Variables

<u>Outcome variable:</u> Individuals with signs of clinical dengue were further tested for laboratory-confirmed evidence of non-structural protein (NS1) of DENV. An individual with positive NS1 results was classified as having dengue. DF is classified in the dataset as non-severe dengue, severe dengue, and dengue with alarming signs. These classifications are closely synonymous with DF, dengue hemorrhagic fever (DHF),

and dengue shock syndrome (DSS) [21]. DF severity was categorized as non-severe dengue and severe dengue. Based on the world health organization's emphasis on critical care for individuals with alarming signs, people with serious dengue or dengue with alarming signs had severe dengue, while those with non-serious dengue were in the 'non-severe dengue' group [22]. Any missing or other classifications were excluded from the analysis.

Exposure variables: A person with IgG positive DF and a current DF diagnosis and IgM positive result were classified as having a secondary infection. Individuals with only IgM positive results were classified as having a primary infection. Infecting DENV serotypes were classified based on polymerase chain reaction RT-qPCR results [23] and was reported in the dataset as DENV-1, DENV- 2, DENV-3, or DENV-4. Demographic variables reported in the dataset include age and sex. Sex was categorized as male or female. Baja California, Baja California Sur, Sonora, Sinaloa, and Chihuahua were defined as the 'Northwest' region. Durango, Coahuila, Nuevo Leon, and Tamaulipas were defined as 'Northeast' region. Zacatecas, San Luis Potosi, San Luis Potosi, Aguascalientes, Guanajuato, Queretaro, Nayarit, Jalisco, Colima, and Michoacan were categorized as 'Center west' region. Hidalgo, Mexico City, Distrito Federal, Morelos, Puebla, Tlaxcala were in the 'Center' region, while Oaxaca, Guerrero, Veracruz, Chiapas, Tabasco, Campeche, Quintana Roo and Yucatan were classified under 'Southeast' region.

Statistical Analysis

Dengue severity was tabulated by demographic and predictor variables. Variances across subgroups were tested for statistical significance using chi-square tests for categorical variables and independent sample t-tests for continuous variables. The Type I error alpha value was set at 0.05 for all significance tests. Univariate ~~analysis was~~ conducted for descriptive purposes, and bivariate analyses were performed to determine associations between different predictors and dengue severity. All bivariate analyses were adjusted for False Discovery Rate (FDR).

Mediation Analysis

A regression-based approach may be used in mediation analysis to assess the effect of binary mediators and binary outcomes. When using the product method in the mediation analysis, the procedure comprises the estimation of two regression equations; equation 1 evaluates the effect of the exposure (x) and covariate (c) on the mediator (m), while equation 2 estimates the effect of x, m and c on the outcome (Y) [24].

$$\text{logit}\{P(M=1|a, c)\} = \beta_o + \beta_1 x + \beta'_2 c \ldots\ldots\ldots\ldots\ldots\ldots\ldots\ldots\ldots \text{(1)}$$

$$\text{logit}\{P(Y=1|x, m, c)\} = \theta_o + \theta_1 x + \theta_2 m + \theta_3 c \ldots\ldots\ldots\ldots\ldots\ldots\ldots\ldots\ldots \text{(2)}$$

where, respectively, $\beta_0 + \beta_1$ and β' represent the intercept and effects of age and confounder on infectivity status. Similarly, θ_0, θ_1, θ_2, θ_3 and represent the intercept and respective effects of age, infectivity status and confounder on disease severity classification.

Age was categorized into a four-level variable due to an established bimodal age pattern among individuals with DENV-2 secondary infection[19]. Age (in years) was categorized as '0-20',' 21-40',' 41-60', '61 and older'. In their paper, Hayes and Preacher introduced the concept of relative indirect, direct total effect as multi-categorical independent variables [25]. To compute this, the independent variable 'age_cat' was dummy coded, with the '0-20' group as the reference. Equations (1) and (2) were therefore transformed into (3) and (4), respectively. That may also be visualized in the path diagram (Figure 2).

$$\text{logit}\{P(M=1|a, c)\} = \beta_o + \beta_1 x_1 + \beta_2 x_2 + \beta_3 x_3 + \beta'_4 c \ldots\ldots\ldots\ldots\ldots\ldots \text{(3)}$$

$$\text{logit}\{P(Y=1|x, m, c)\} = \theta_o + \theta_1 x_1 + \theta_2 x_2 + \theta_3 x_3 + bm + \theta'_4 c \ldots\ldots\ldots\ldots\ldots \text{(4)}$$

The relative direct effect of age (x) on disease severity (y) is captured by the θ_i estimates. In contrast, the relative indirect effect of x on y through infection status (m) is given by the products β_i b, where i = 1,2,3. The word 'relative' is used to emphasize that the effects are relative to the reference group '0-20' and

would change if the reference group were substituted [25]. Since the independent variable has multiple categories, the total effect was estimated by the parameter estimates from the dummy coded groups and given by equation (5). The estimated produced by the mean differences between the age groups are referred to as relative total effects. The total relative effect may also be calculated by summing up the relative direct and indirect effects (6).

$$\text{logit}\ \{P\ (Y = 1|x, c)\} = \phi_o + \phi_1 x_1 + \phi_2 x_2 + \phi_3 x_3 + \phi'_4 c \ldots\ldots\ldots\ldots (5)$$

$$\phi_i = \theta_i + \beta_i b \ldots\ldots\ldots\ldots\ldots\ldots\ldots\ldots (6)$$

Based on the directed acyclic graph (DAG) (Figure 1), ‘region’ was explored as a potential random effect in a two-level hierarchical model for the association between age and disease severity. The Wald test for the region for random effect was insignificant, and its effect in a fixed-effect model was ~~neither significant~~. For each model, sex and DENV serotype were used as covariates. Based on the Akaike Information Criterion (AIC) and likelihood ~~ration~~ test, potential confounders and interactions were explored, and the model without ~~interaction~~ was found to be the best model.

Spatial Analysis

Severe and non-severe dengue cases were summed up by secondary infection status. That was used in a cluster analysis where the spatial variability of severe dengue was compared by infection status across Mexican municipalities. The spatial distribution of severe and non-severe dengue was visualized for both primary and secondary infection. An optimized outlier analysis of severe dengue was then performed for each infection status. Statistically significant spatial clusters of high values (hot spots) and low values (cold spots) were identified using the Anselin Local Moran's I (LMi Index) statistic. The FDR correction method was used at a 5% error rate to correct for multiple testing and spatial dependence [26].

Coding and Environment

Preprocessing of data, analysis, and generation of figures were done using SAS (version 9.4, SAS Institute Inc., Cary, NC, USA).

Results

Of the 52,212 individuals with reported data, 60% were female, with roughly half (50%) living in the Southeast region and 81.2% having non-severe dengue (Table 1). The average age was 32 years, ranging from 0 to 100 years, with females being averagely older (33 years) than males (30 years). The difference between primary and secondary infection by gender was not ~~statically~~ significant ($\square^2$ = 0.007, p= 0.9447 (Table S1). On average, individuals with secondary infection were older than those with primary infection (Figure S1). This difference was statistically significant [$t_{(7961.7)} = -15.99$, $p < 0.0001$]. The prevalence of secondary infection was higher among individuals with severe dengue (95%) than those with non-severe dengue (87%). Descriptive statistics for the age category are in Table 2. A chi-square assessment revealed statistically significant differences between age categories for infection status ($\square^2$= 211.4, <0.0001) and disease severity ($\square^2$= 178.0, < 0.0001). Increasing age was associated with higher proportions of secondary infection, while the extreme age categories (youngest and oldest) were significantly associated with higher proportions of disease severity.

Table 1: Demographic characteristics by dengue classification

Parameter		Dengue Classification, n (%)		
		Severe Dengue	Non-Severe Dengue	All
**Sex	Male	4645 (31.42%)	16243 (77.76%)	20888 (40.01%)
	Female	5181 (16.54%)	26143 (83.46%)	31324 (59.99%)
**Region	Center	479 (4.87%)	2156 (5.09%)	2635 (5.05%)
	Center- West	966 (9.83%)	6855 (16.17%)	7821 (14.98%)
	North - East	1173 (11.94%)	11100 (26.19%)	12273 (23.51%)
	North - West	435 (4.43%)	2837(6.69%)	3272 (6.27%)
	South - East	6773 (68.93%)	19438 (45.86%)	26211 (50.20%)
^{NS} Age in years (SD)		32 (±19.80)	32 (±18.71)	32 (±18.92)
Total (n)		9826 (18.18%)	42386 (81.18%)	52212 (100%)

** Chi-square performed for group differences had p <0.001

^{NS} P-value of T-test not significant

Table 2: Descriptive statistics for age categories stratified by infection status and disease severity

	Infection Status		Chi-square (p-value)	Disease Severity		Chi-square (p-value)
Age Category (years)	Secondary Infection	Primary Infection	χ^2= 211.4, <0.0001	Severe Dengue	Non-Severe Dengue	χ^2= 178.0, < 0.0001
0- 20	14771 (85.7%)	2471 (14.3%)		3414 (19.8%)	13820 (80.2%)	
21 – 40	16617 (89.1%)	2042 (10.9%)		3329 (17.9%)	15317 (82.4%)	
41 – 60	10319 (89.0%)	1275 (11.0%)		2034 (17.5%)	9549 (82.4%)	
61 and over	4401 (92.5%)	357 (7.5%)		1050 (22.1%)	3700 (77.9%)	

There was a lower frequency of severe and non-severe dengue among individuals about 0-5 years for those with secondary infection compared to older individuals (Figure S2a&b). The average frequency of dengue among those with secondary infection from about five years to 60 years was about 20% and 70%, respectively, for those with severe and non-severe dengue. However, above 60 years, there was an increase in the frequency among those with severe dengue than those with non-severe dengue (Figure S2a&b). The frequency of non-severe dengue decreased with age among those with primary infection. However, the frequency of severe dengue among those with primary infection and severe dengue remained around 5% for the entire age distribution except for those above 60 years who had higher frequencies of severe dengue (Figure S2a&b). Figure S3a &b show variations in the frequency of primary and secondary infection from 2012 to 2017 for severe and non-severe dengue. For those with primary infection, there is an observed increase in the proportion of severe and non-severe dengue between 2016 and 2017. Although secondary infection was commonest in the Southeast region (55.8%), primary infection was commonest in the Northeast region (80.5%) (Table S1 & Figure S4). Regional differences were statistically significant [χ^2 = 12775.3, p< 0.0001].

DENV-2 was the commonest serotype among individuals with severe and non-severe dengue (Table 3). The serotype distribution pattern was similar for both sexes (Figure S5). However, the proportion of those with secondary infection who had severe dengue was higher than those with non-severe dengue.

Observed serotype and infection status differences were statistically significant (Table 3). The Southeast region had the highest proportion of DENV -2, DENV-3, and DENV-4 and the second highest for DENV-1 (Table S2). Apart from the Southeast region, the Northeast had the highest proportion of DENV-3 and a higher proportion of DENV-4. In contrast, the Center west region had higher proportions of DENV-1 and DENV-2 than the other regions.

Table 3: Proportions and p-value for different chi-squared test results with 95% significance

Parameter		Dengue Classification (%)		
		Severe Dengue	Non-Severe Dengue	Chi-square p-value
Serotype	DENV- 1	58 (0.59%)	233 (0.55%)	<.0001
	DENV- 2	183 (1.86%)	289 (0.68%)	
	DENV- 3	5 (0.05%)	18 (0.04%)	
	DENV- 4	3 (0.03%)	37 (0.09%)	
	Missing	9577 (97.47%)	41809 (98.64%)	
Secondary infection	Yes	9333 (94.98%)	36735 (86.67%)	<.0001
	No	493 (5.02%)	5651 (13.33%)	

Compared with those '0-20 years', individuals '21 to 40 years',' 41-60 years' and '61 years and over' had 128%, 190% and 443% higher odds of having a secondary infection when holding sex and serotype status constant (Table 4). The relative indirect effects of the age categories on disease severity were calculated by multiplying the β_i coefficients with *b* (Figure S6) and are shown in equations (7), (8), and (9).

$$\beta_1 * b = 0.8238 \times 1.2931 = 1.065 \quad \ldots \quad (7)$$

$$\beta_2 * b = 1.0663 \times 1.2931 = 1.379 \quad \ldots \quad (8)$$

$$\beta_3 * b = 1.6927 \times 1.2931 = 2.189 \quad \ldots \quad (9)$$

Compared to those within the '0-20 years' age range, individuals within the '21-40 years' [0.82, (CI: 0.77, 0.88)] and '41-60 years' [0.82, (CI: 0.76, 0.89)] age groups had lower odds of severe dengue, while those in the '61 years and over' group [1.19, (CI: 1.05, 1.35)] had higher odds of severe dengue. The effect of age on dengue severity was no longer significant after introducing infectivity status into the logistic regression

model (Table 4). While the total effect for the first two group comparisons (X_1 and X_2) were smaller (10 and 11) than the indirect effects, the total effect for X_3 (12) indicated a higher effect after summing up the direct and indirect effects.

Table 4: Estimated coefficients from equations (3) – (5), estimated in SAS using indicator coding with statistical errors in parenthesis.

Outcome	M			Y					
		Coefficient (SE)	OR [CI]		Coefficient (SE)	OR [CI]		Coefficient (SE)	OR [CI]
Indicator coding									
Constant	βo	-1.8350 (0.2047) *		ϕ_o	-1.*4923* (0.0292) *		θ_o	-1.8277 (0.2131) *	
'21-40' *vs* '0-20' **(X_1)**	$\beta 1$	0.8238 (0.1830) *	2.28 [1.59, 3.26]	ϕ_1	-0.1959 (0.0324) *	0.82 [0.77, 0.88]	θ_1	-0.0644 (0.1963) ^{NS}	0.94 [0.64, 1.38]
'41-60' *vs* '0-20' **(X_2)**	$\beta 2$	1.0663 (0.2114) *	2.90 [1.92, 4.40]	ϕ_2	-0.1969 (0.0408) *	0.82 [0.76, 0.89]	θ_2	-0.1639 **(**0.2320**)** ^{NS}	0.85 [0.54, 1.34]
'61 and over' *vs* '0-20' **(X_3)**	$\beta 3$	1.6927 (0.3474) *	5.43 [2.75, 10.74]	ϕ_3	0.1757 (0.0641) *	1.19 [1.05, 1.35]	θ_3	-0.0754 (0.3491) ^{NS}	0.93 [0.47, 1.84]
Infection status **(M)**							b	1.2931 (0.1725) *	3.64 [2.60, 5.11]

$^{+}$ P-value of significance test < 0.0001
^{NS} P-value not significant

Total effect (X_1) = 1.065 - 0.1959 = 0.8691 ………………………………………… (10)

Total effect (X_2) = 1.379 - 0.1969 = 1.1821 ………………………………………… (11)

Total effect (X_3) = 2.189 + 0.1757 = 2.3647 ………………………………………… (12)

Hotspots for those with severe dengue attributable to primary infection were primarily found in the northeastern region, while secondary infection hotspots were mainly in the southeastern region (Figures S7 a and b). The Northeast region had more hotspots for severe dengue among those with primary infection. Furthermore, when compared to all regions (other than the Southeast), DENV-3 was most predominant in the northeast region.

Discussion

The effect of age is primarily mediated by secondary infection status. That indicates that assessing an individual's risk for secondary infection needs to be prioritized during dengue prevention initiatives. Notably, individuals under five years and older than 60 need critical management of dengue infection. Two things common to the Northeastern region between 2012 and 2017 were the relatively higher prevalence of DENV-3 compared to other serotypes and the presence of severe dengue hotspots among individuals with primary infection. While efforts must be taken to halt the secondary transmission of DF, rigorous measures in areas newer to DF spread must be considered to address DF complications. Surveillance among dengue-naïve populations should pay particular attention to the introduction of DENV-3 serotypes.

Children below 14 years and adults older than 50 years have been found to have a higher death and hospitalization rate [19]. A similar trend was seen in our study population among individuals with secondary infection, where the frequency of dengue severity slightly decreases from about five years and increases again from 60. The association between higher age and secondary infection is consistent with the literature [27]. Furthermore, the association between severe dengue and ageing populations [27] may be explained by the higher propensity of exposure to a second infection as a person ages. When an individual develops ADE from initial infection [28], cross-reaction with the second infecting virus results in immune complexes that result in cytokine release later in the infection process, further leading to vascular permeability, shock, and increasing the possibility of death [28].

Characterization of DENV-2 in the Southeastern region has been associated with the American/Asian genotypes from Southeast Asia [29]. Since the Southeastern region had the highest proportion of DENV-2, DENV-3, and DENV-4 serotypes, the association between the southeastern region and dengue severity hotspots among those with secondary infection may be attributable to the prevalence of old circulating genotypes of the DENV-2, DENV-3, and DENV-4 serotypes in the region.

Apart from the Southeastern region, DENV-3 was also prevalent in the Northeastern region, and the association between the northeastern region and severe dengue among those with primary infection suggests a possible link between the predominantly circulating serotype and disease severity. DENV-3 has

been associated with severe dengue for individuals with primary infection, while secondary infection with DENV-1, DENV-2, and DENV-4 are primarily associated with severe dengue [30]. Furthermore, the significant clustering of dengue severity among those with primary infection in the Northwestern region may be related to the migration of DENV serotypes towards the north as climate change may favor *Aedes aegypti* dissemination towards central and northern regions [31].

The association between increasing age and dengue severity among individuals with both primary and secondary infection has been established [1]. However, in our study, this was particularly true for individuals older than 60. After controlling for sex, serotype and age, individuals with secondary infection had 3.6 [CI: (2.599, 5.110)] times the odds of having severe dengue. Furthermore, after adding infectivity status into the model, the relationship between age and disease severity was no longer significant. This study shows that, rather than a linear relationship, extreme age groups are at an increased risk of severe dengue and this relationship is largely mediated by infectivity status. While age would indicate more years at risk and an increased likelihood of severe infection, it has been suggested that infants within 6-12 months may be at risk of severe dengue due to placental transfer of maternal IgG [32].

This study has several limitations. First, some studies have linked the history of two or more previous infections with a decreased risk of a new infection [8]. This hypothesis could not be tested in our population since information about several prior dengue infections in the past was unavailable. Secondly, there was a vast amount of missingness, particularly for the serotype data. However, the final sample size was 52,212, and 827 serotype records were available for analysis, with 9577 missing severe dengue (97.47%) classification and 41809 missing non-severe dengue (98.64%) classification (Table 3). Missingness was assumed to be random since the proportion of missingness across disease severity and covariates were proportional, and a complete case analysis was performed for all regression models.

Conclusion

Understanding the ~~causal pathway and mechanism~~ with which age affects dengue severity allows targeting the right population group for interventions. The mediation effect of secondary infection status on

dengue severity will enable scientists to focus on ways to prevent dengue re-infection and active management of patients with prior history of dengue, as well as in dengue-naïve populations. Primary infection in Northeastern Mexico and secondary infection in other regions of Mexico require continued surveillance. Furthermore, through the indirect effect of secondary infection, the aged population are at an increased risk of dengue fever complications and requires critical care and management.

References.

1. Thai KT, Nishiura H, Hoang PL, Tran NT, Phan GT, Le HQ, et al. Age-specificity of clinical dengue during primary and secondary infections. PLoS Negl Trop Dis. 2011;5(6):e1180. Epub 2011/06/30. doi: 10.1371/journal.pntd.0001180. PubMed PMID: 21713018; PubMed Central PMCID: PMCPMC3119638.
2. Gibbons RV, Vaughn DW. Dengue: an escalating problem. Bmj. 2002;324(7353):1563-6.
3. Thai KT, Cazelles B, Nguyen NV, Vo LT, Boni MF, Farrar J, et al. Dengue dynamics in Binh Thuan province, southern Vietnam: periodicity, synchronicity and climate variability. PLoS neglected tropical diseases. 2010;4(7):e747.
4. Musso D, Rodriguez-Morales AJ, Levi JE, Cao-Lormeau VM, Gubler DJ. Unexpected outbreaks of arbovirus infections: lessons learned from the Pacific and tropical America. Lancet Infect Dis. 2018;18(11):e355-e61. Epub 2018/06/24. doi: 10.1016/s1473-3099(18)30269-x. PubMed PMID: 29934112.
5. Nealon J, Bouckenooghe A, Cortes M, Coudeville L, Frago C, Macina D, et al. Dengue Endemicity, Force of Infection, and Variation in Transmission Intensity in 13 Endemic Countries. The Journal of Infectious Diseases. 2020;225(1):75-83. doi: 10.1093/infdis/jiaa132.
6. Laserna A, Barahona-Correa J, Baquero L, Castañeda-Cardona C, Rosselli D. Economic impact of dengue fever in Latin America and the Caribbean: a systematic review. Rev Panam Salud Publica. 2018;42:e111. Epub 2019/05/17. doi: 10.26633/rpsp.2018.111. PubMed PMID: 31093139; PubMed Central PMCID: PMCPMC6386068.
7. Herrera-Martinez AD, Rodríguez-Morales AJ. Potential influence of climate variability on dengue incidence registered in a western pediatric Hospital of Venezuela. Trop Biomed. 2010;27(2):280-6. Epub 2010/10/22. PubMed PMID: 20962726.
8. Amaya-Larios IY, Martínez-Vega RA, Diaz-Quijano FA, Sarti E, Puentes-Rosas E, Chihu L, et al. Risk of dengue virus infection according to serostatus in individuals from dengue endemic areas of Mexico. Scientific Reports. 2020;10(1):19017. doi: 10.1038/s41598-020-75891-z.
9. Kyle JL, Harris E. Global spread and persistence of dengue. Annu Rev Microbiol. 2008;62:71-92.
10. Lee I-K, Hsieh C-J, Lee C-T, Liu J-W. Diabetic patients suffering dengue are at risk for development of dengue shock syndrome/severe dengue: Emphasizing the impacts of co-existing comorbidity(ies) and glycemic control on dengue severity. Journal of Microbiology, Immunology and Infection. 2020;53(1):69-78. doi: https://doi.org/10.1016/j.jmii.2017.12.005.

11. Nunes PC, Sampaio SA, da Costa NR, de Mendonça MC, Lima Mda R, Araujo SE, et al. Dengue severity associated with age and a new lineage of dengue virus-type 2 during an outbreak in Rio De Janeiro, Brazil. J Med Virol. 2016;88(7):1130-6. Epub 2016/04/12. doi: 10.1002/jmv.24464. PubMed PMID: 27061403.

12. Pongpan S, Wisitwong A, Tawichasri C, Patumanond J, Namwongprom S. Development of dengue infection severity score. ISRN Pediatr. 2013;2013:845876-. doi: 10.1155/2013/845876. PubMed PMID: 24324896.

13. Anders KL, Nguyet NM, Chau NV, Hung NT, Thuy TT, Lien le B, et al. Epidemiological factors associated with dengue shock syndrome and mortality in hospitalized dengue patients in Ho Chi Minh City, Vietnam. The American journal of tropical medicine and hygiene. 2011;84(1):127-34. Epub 2011/01/08. doi: 10.4269/ajtmh.2011.10-0476. PubMed PMID: 21212214; PubMed Central PMCID: PMCPMC3005500.

14. Xu C, Pang J, Hsu JP, Leo YS, Lye DCB. Differences in clinical features and dengue severity between local and migrant Chinese with dengue infection in Singapore. PLoS One. 2018;13(8):e0201441. Epub 2018/08/16. doi: 10.1371/journal.pone.0201441. PubMed PMID: 30110348; PubMed Central PMCID: PMCPMC6093606.

15. Focks D, Barrera R. Dengue Transmission Dynamics: Assessment And Implications For Control. WHO Report of the Scientific Working Group meeting on Dengue, Geneva. 2006.

16. Egger JR, Coleman PG. Age and clinical dengue illness. Emerging infectious diseases. 2007;13(6):924-5. doi: 10.3201/eid1306.070008. PubMed PMID: 17553238.

17. Khetarpal N, Khanna I. Dengue Fever: Causes, Complications, and Vaccine Strategies. J Immunol Res. 2016;2016:6803098. Epub 2016/08/16. doi: 10.1155/2016/6803098. PubMed PMID: 27525287; PubMed Central PMCID: PMCPMC4971387.

18. Morens DM, Fauci AS. Dengue and Hemorrhagic FeverA Potential Threat to Public Health in the United States. JAMA. 2008;299(2):214-6. doi: 10.1001/jama.2007.31-a.

19. Guzmán MG, Kouri G, Bravo J, Valdes L, Susana V, Halstead SB. Effect of age on outcome of secondary dengue 2 infections. International Journal of Infectious Diseases. 2002;6(2):118-24. doi: https://doi.org/10.1016/S1201-9712(02)90072-X.

20. Fried JR, Gibbons RV, Kalayanarooj S, Thomas SJ, Srikiatkhachorn A, Yoon IK, et al. Serotype-specific differences in the risk of dengue hemorrhagic fever: an analysis of data collected in Bangkok, Thailand from 1994 to 2006. PLoS Negl Trop Dis. 2010;4(3):e617. Epub 2010/03/09. doi: 10.1371/journal.pntd.0000617. PubMed PMID: 20209155; PubMed Central PMCID: PMCPMC2830471.

21. Phanitchat T, Zhao B, Haque U, Pientong C, Ekalaksananan T, Aromseree S, et al. Spatial and temporal patterns of dengue incidence in northeastern Thailand 2006–2016. BMC Infectious Diseases. 2019;19(1):743. doi: 10.1186/s12879-019-4379-3.

22. Srikiatkhachorn A, Rothman AL, Gibbons RV, Sittisombut N, Malasit P, Ennis FA, et al. Dengue--how best to classify it. Clinical infectious diseases : an official publication of the Infectious Diseases Society of America. 2011;53(6):563-7. Epub 2011/08/10. doi: 10.1093/cid/cir451. PubMed PMID: 21832264.

23. Secretaría de Salud M. LINEAMIENTOS PARA LA VIGILANCIA POR LABORATORIO DEL DENGUE Y OTRAS ARBOVIROSIS, INDRE (INSTITUTO DE DIAGNÓSTICO Y REFERENCIA EPIDEMIOLÓGICOS ‘DR. MANUEL MARTÍNEZ BÁEZ’). 2021. Available from: https://www.gob.mx/cms/uploads/attachment/file/629265/Lineamientos_Dengue_Arb_V1-2021.pdf.

24. VanderWeele TJ. Mediation Analysis: A Practitioner's Guide. Annu Rev Public Health. 2016;37:17-32. Epub 2015/12/15. doi: 10.1146/annurev-publhealth-032315-021402. PubMed PMID: 26653405.

25. Hayes AF, Preacher KJ. Statistical mediation analysis with a multicategorical independent variable. Br J Math Stat Psychol. 2014;67(3):451-70. Epub 2013/11/06. doi: 10.1111/bmsp.12028. PubMed PMID: 24188158.

26. Pro A. Optimized Outlier Analysis (Spatial Statistics) n.d [1/27/2022]. Available from: https://pro.arcgis.com/en/pro-app/latest/tool-reference/spatial-statistics/optimizedoutlieranalysis.htm.

27. Thai KTD, Nishiura H, Hoang PL, Tran NTT, Phan GT, Le HQ, et al. Age-specificity of clinical dengue during primary and secondary infections. PLoS neglected tropical diseases. 2011;5(6):e1180-e. Epub 2011/06/21. doi: 10.1371/journal.pntd.0001180. PubMed PMID: 21713018.

28. Guzman MG, Alvarez M, Halstead SB. Secondary infection as a risk factor for dengue hemorrhagic fever/dengue shock syndrome: an historical perspective and role of antibody-dependent enhancement of infection. Arch Virol. 2013;158(7):1445-59. Epub 2013/03/09. doi: 10.1007/s00705-013-1645-3. PubMed PMID: 23471635.

29. Cisneros A, Díaz-Badillo Á, Cruz-Martínez G, Tovar R, Ramírez-Palacios LR, Jiménez-Rojas F, et al. Dengue 2 genotypes in the state of Oaxaca, Mexico. Archives of Virology. 2006;151(1):113-25.

30. Soo KM, Khalid B, Ching SM, Chee HY. Meta-Analysis of Dengue Severity during Infection by Different Dengue Virus Serotypes in Primary and Secondary Infections. PLoS One. 2016;11(5):e0154760. Epub 2016/05/24. doi: 10.1371/journal.pone.0154760. PubMed PMID: 27213782; PubMed Central PMCID: PMCPMC4877104.

31. Mweya CN, Kimera SI, Stanley G, Misinzo G, Mboera LE. Climate Change Influences Potential Distribution of Infected Aedes aegypti Co-Occurrence with Dengue Epidemics Risk Areas in Tanzania. PLoS One. 2016;11(9):e0162649. Epub 2016/09/30. doi: 10.1371/journal.pone.0162649. PubMed PMID: 27681327; PubMed Central PMCID: PMCPMC5040426.

32. Centers for Disease Control and Prevention. Dengue: Clinal Presentation 2021 [7/26/2022]. Available from: https://www.cdc.gov/dengue/healthcare-providers/clinical-presentation.html.

Click here to access/download;Figure;Figure_1.pdf

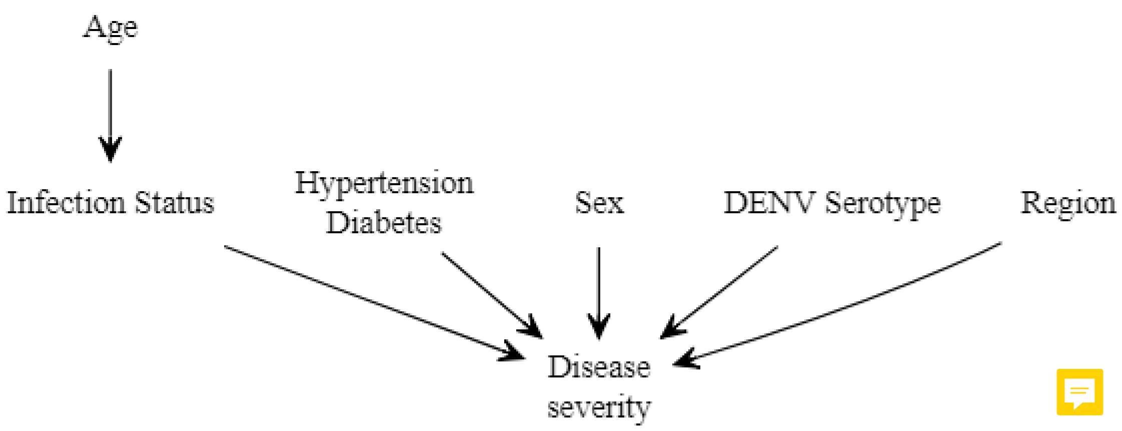

Click here to access/download;Figure;Figure_2.pdf

	Indicator Coding			
	0-20 (Control)	21-40	41-60	61 or older
X1	0	1	0	0
X2	0	0	1	0
X3	0	0	0	1

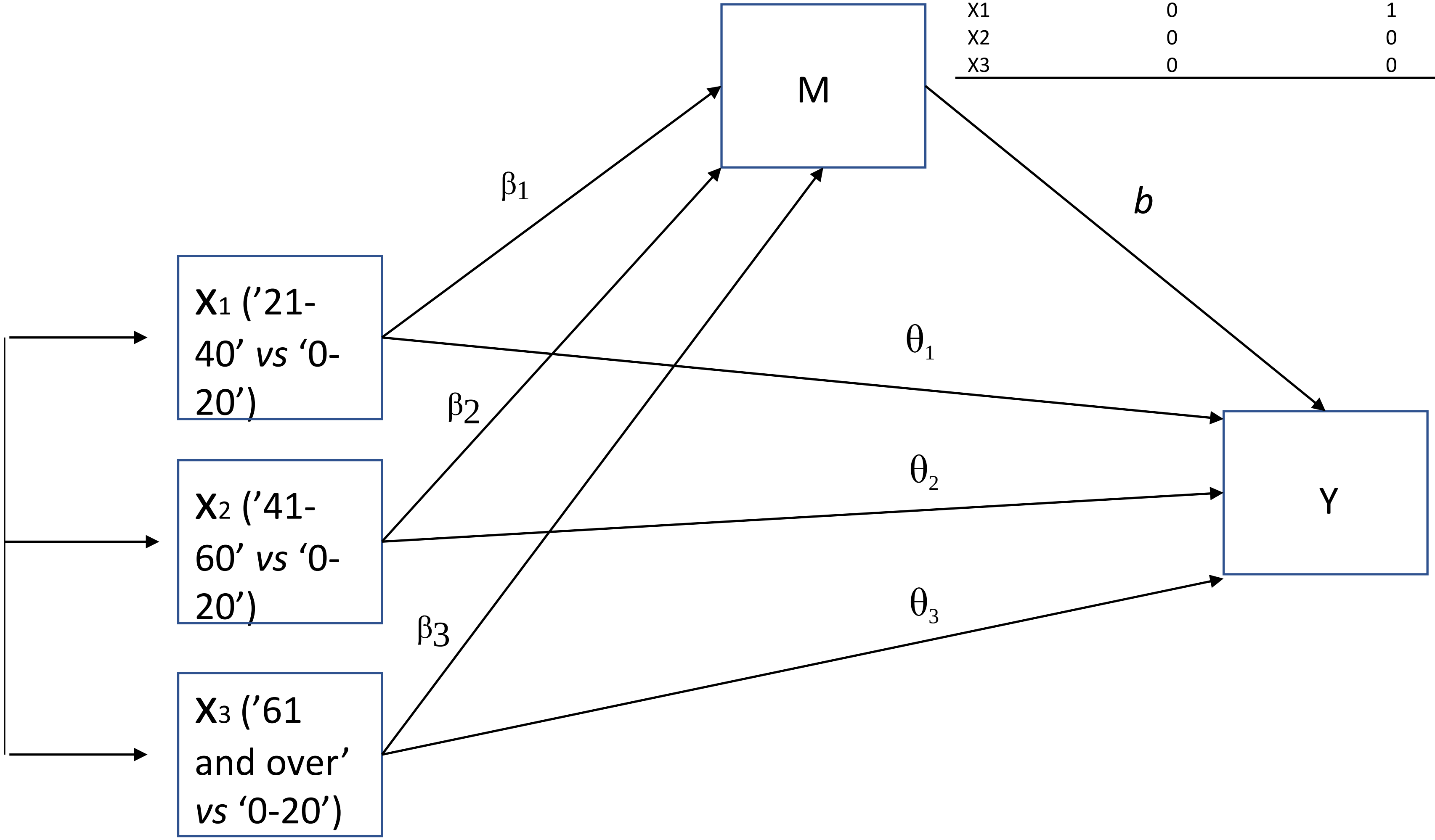

Click here to access/download
Supporting Information - Compressed/ZIP File Archive
Supplementary_figures_and_tables.zip
