# Supplementary material for: Direct and indirect effects of age on dengue severity: The mediating role of secondary infection
Source: PLoS Negl Trop Dis. 2023 Aug 9;17(8):e0011537. doi: 10.1371/journal.pntd.0011537 (PMC10441797; doi:10.1371/journal.pntd.0011537)
Supplement: Attachment — Submitted filename: reponse_to_reviewers_plos_ntd.docx [file pntd.0011537.s013.docx]

PNTD-D-22-01621

Dear *PLOS NTD editor* and peer reviewers,

We offer our sincere thanks for your thoughtful reviews of our manuscript, “Direct and indirect effects of age on dengue severity: the mediating role of secondary infection’. We made numerous revisions in accordance with your feedback. Your comments, our responses, and information about the changes are described in point-by-point detail below. We also uploaded a version of the manuscript in which all changes have been highlighted in yellow.

Comments and responses:

Reviewer #1

METHODS.

**Comment 1. The sample size is sufficient to ensure adequate power to address the hypothesis test. However, some questions need to be clarified. The author needs to check the equations in the mediation analysis. In line 169, the author wrote the total relative effect may also be calculated by summing up the relative direct and indirect effects (6) фi = θi + ᵦi b. However, the author calculated the total effect in equations (10-12) as Total effect = фi + ᵦi b. For example, - 0.1959 was ф1 not θ1 in equation 10. And the фi ≠ θi + ᵦi b in table 4. Maybe I am misunderstanding how these models work, but then I think it would be helpful to walk the reader through this a bit more.**

Response: We really appreciate that you pointed out this critical error. Beginning **line 289, page 12**, the calculation of the total effect uses **θi,** rather than **фi** as the relative indirect effect for the total effect calculation.

**Comment 2. There should also be a line between age and disease severity in Figure 1. Because according to existing information, secondary infection is not a complete mediator variable between age and severe dengue.**

Response: Thank you for pointing out this observation. We have updated Figure 1 to include a line from age to disease severity.

RESULTS.

**Comment 1: What did Percent of Total Frequency mean in Fig S2&S3?**

Response: Thank you for this clarification question. The percentages add up to 100% for each age and time point. Hence the percentages for primary and secondary infection for non-severe dengue plus the percentages of primary and secondary infection for non-severe dengue add up to 100 for those 0 years old. To improve clarity, starting from **line 243, page 10**, the sentences ‘*Figures S2 and S3 show the proportions of severe (a) and non-severe (b) dengue and the proportions of infection status for each category. Hence*, *all four proportions add up to 100% for every age. Compared to the other ages, there was a lower frequency of secondary infection among individuals about 0-5 years for both severe and non-severe dengue (Figure S2a&b). The average frequency of dengue among those with secondary infection from about five years to 60 years was about 20% and 70%, respectively, for those with severe and non-severe dengue. However, above 60 years, there was a steeper increase in the frequency among those with severe dengue than those with non-severe dengue (Figure S2a&b).*’ have been modified.

**Comment 2. It is recommended to do statistical tests in Fig S2&S3, such as trend tests and comparison tests between groups.**

Response: We appreciate you sharing this recommendation. Beginning **line 253, page 10**, the sentence “*Differences observed between primary and secondary infection for severe dengue (t-value= -2.56, p=0.001) was significant but not for non-severe dengue (t-value=-0.99, p=0.3219). Figures S3a &b show variations in the frequency of primary and secondary infection from 2012 to 2017 for severe and non-severe dengue. The Cochrane-Armitage trend test for the difference between primary and secondary infection for severe dengue (Z = 2.01, p= 0.04) and non-severe dengue (Z= -14.67, p<0.0001) were both statistically significant.*” have been added to reflect the t-test and Cochrane-Armitage trend tests done.

**Comment 3. Figure S7 was not found.**

Response: Thank you for noting the missing figure. It has been uploaded with the other files in this round.

CONCLUSIONS.

**Comment: The authors should carefully check the results of the mediation analysis.**

Response: Thank you. In addition to the changes done to address previous comments, beginning from **line 272, page 11**, there have been further edits to the results. This also reflects different estimates because of the change of the reference group to ‘0-1’ years.

SUMMARY AND GENERAL COMMENTS
**Comment: The authors have attempted to answer an important question about severe dengue:  if the effect of age on dengue severity was mediated by infectivity status? While the intention is nice, there are several issues that may influence whether the paper is worth publishing.**

Response: Thank you for all your valued comments and suggestions. We have addressed them based on all the recommendations we received.

EDITORIAL AND DATA PRESENTATION MODIFICATIONS

**Comment 1. According to existing studies, the elderly and children are high-risk groups for dengue fever. If the control group is set as young people, the results may be more robust.**

Response: We appreciate that you raised this important point. Further categorizing the age groups based on prior studies, we decided to make the control group individuals less than 1 year old, anticipating that most severe cases occurred around the 6-10 month age bracket [1]. We also kept the elderly as those 60 years or older [2]. Beginning **line 134, page 5,** the sentence ‘*Age (in years) was categorized as '0-1','> 1-20','> 20-60', '>60' to account for the infant and elderly populations that have a higher risk for severe dengue [1, 2].*’ has been added to the methods section.

**Comment 2 & 3. Much of the article described the regional differences of severe dengue, but why did the author not adjust it in the model? Some factors, such as education, income, diabetes, and hypertension, that may affect the results should also be controlled for in the mediation analysis.**

Response: Thank you for pointing this out. After re-running the model, the variables available in the dataset, which includes region, diabetes, and hypertension were controlled for in the model.

Reviewer #2

METHODS

**Comment 1: The biggest question I have (which I think would help with the rest of the paper), is: why was a mediation analysis chosen to answer the research question of interest? Instead of finding the direct and indirect effects of age on dengue severity through secondary infection status, it sounds more like the research group is trying to see how age moderates the effect of secondary infection status on dengue severity. I would consider a moderation analysis, instead. If not, I would suggest making more explicit 1) the research question of interest, and hypothesized answers for that question; and 2) why a mediation analysis was chosen to answer this research question.**

Response: Thank you for this clarification question and suggestion. We have stated more clearly what our research question and hypotheses are. Beginning **line 100, page 4**, the following sentences have been modified as follows: ‘*Hence, our study aimed to quantify the direct and indirect effect of age on severe dengue through the mediating role of secondary infection (Figure 1). This mediation analysis was performed to help provide information about the mechanism with which age predicts dengue severity. We hypothesized that the effect of age on dengue severity would be insignificant after the introduction of infectivity status into a model predicting severe dengue, after controlling for confounding variables. Further, we hypothesized that the effect of infectivity status would be moderated by DENV serotype. ’*

**Comment 2: I would suggest making more explicit 1) the population of interest, 2) how the sample was drawn from this population.**

Response: Thank you for this suggestion. Beginning **line 115, page 4** we have added the sentence “*Our population of interest included all individuals diagnosed with dengue in Mexico with information about IgM and IgG serology results. The dataset had information about individuals meeting these criteria from 2012 to 2017.”.*

**Comment 3: The sample size was not mentioned in the Methods section (I would suggest doing that there); however, given the large N (approximately 52,000), I would think there is sufficient power for this analysis. I would suggest a sentence confirming this.**

Response: Thank you for pointing this out. Beginning **line 158, page 6** the sentence ‘*The final sample size for the analysis was 52212.’* has been added.

**Comment 4: Additionally, there was little mention of the Spatial Analysis (e.g., in the Introduction, it was not mentioned), and the rationale for including it was not clear.**

Response: Beginning **line 106, page 4** we have added more information about the spatial analysis and our hypothesis: “*We further characterized the spatial distribution of infectivity status for severe dengue across Mexico. While a secondary infection of dengue may be due to a homologous infection, a secondary infection that is associated with severe dengue is likely due to infection with a heterologous DENV serotype[3]. Hence, we expected to find spatial differences across infectivity status for those with severe dengue. Regional differences of infectivity may require extra vigilance and efforts to control dengue in areas with significant clusters of secondary infection.”*

RESULTS.

**Comment 1: The analysis presented matches the analysis plan; however, although the authors hoped to estimate direct and indirect effects, estimates of these effects were not explicitly presented.**

Response: As suggested, we have presented the estimates more explicitly in a figure. This addresses your third comment on the results section as well.

**Comment 2: The figures were not numbered or labeled. I would suggest adding captions to all figures and tables.**

Response: Thank you. The figure legend has been added to main manuscript, beginning **line 366, page 15**.

**Comment 3: I would suggest adding a figure that illustrates the results.**

Response: Thank you. We have addressed this in comment 1.

CONCLUSIONS

**Comment: There is a statement that the effect of age on dengue severity is primarily mediated by secondary infection status. I'm not sure the authors can say this unless they have the percentage of the total effect that is due to the indirect effect, and that percentage is substantially greater than 50%; I did not see that anywhere in the paper.**

Response: We have modified the verbiage based on your recommendation. Beginning **line 303, page 13** the sentence now reads ‘*Individuals between 0-1 year and those 60 years and older have relatively higher odds of severe dengue and need critical management of dengue infection. However, the effect of age on severe dengue appears to be no longer significant after introducing secondary infection status into the causal model estimating the effect of age on severe dengue*.’

SUMMARY AND GENERAL COMMENTS
**Comment: I think this article has potential to contribute to the dengue literature and public health research at large; however, I think much work needs to be done on defining the research question, being explicit about the population and sample of interest, choosing an analysis (or analyses) that answers that research question, and presenting results that correspond to the chosen analysis.**

Response: Thank you once again for all your feedback and suggestions. On page 4, from **lines 100 to 111**, this comment has been addressed.

Reviewer #3

METHODS

**Comment 1: The objective of the study is clearly stated with a testable hypothesis stated. The study design using a cross-sectional method is appropriate for the objectives. The population has been clearly defined and described with adequate sample sizes. The statistical analysis used to support conclusions are appropriately conducted. Since secondary data from public database was used there is no concern regarding ethical or regulatory requirements.**

Response: Thank you. We appreciate your feedback.

Reviewer #4

INTRODUCTION

**Line 88/89 - I suggest using the current classification of dengue cases and not the old one in this part of the introduction section: “Of the people with a secondary infection, 2% - 3% progress to dengue hemorrhagic fever (DHF), which may further result in dengue shock syndrome (DSS) and death [17].”**

Response: Thank you for this comment. Beginning **line 88, page 3,** the sentence now reads ‘*Of the people with a secondary infection, 2% - 3% progress to dengue with warning signs, which may further result in severe dengue and death [4]*.’

METHODS
**The description of spatial analysis is not well-detailed. They used the Anselin Local Moran's I (LMi Index) statistic, but it is not clear how this method works. I suggest to the authors to describe this method more carefully.**

Response: We appreciate this recommendation. To explain the Anselin Local Moran’s I statistic, beginning **line 211, page 8** the following sentence has been added ‘*The Anselin local Moran’s I is a local indicator of spatial association (LISA). The LISA for an observation shows the extent to which there is significant spatial clustering of similar values around that observation, while the sum of all LISAs correlated with the global spatial association [5]. The statistics behind the Anselin formula is further explained elsewhere [6]*.’

RESULTS

**The authors did not show the clusters results and just added a comment in the discussion section. They mentioned municipalities in the method but presented analysis by regions (tables). I consider necessary to present a figure (map) to visualize the clusters. This will provide to the readers a better understanding about the distribution pattern in the study area. If possible, I also suggest presenting a map showing the distribution of cases by serotypes (DENV1, DENV2, DENV3 and DENV4).**

Response: Thank you for these comments.

1. Cluster results are presented beginning **line 292, page 13** and it reads ‘*Clusters for those with severe dengue attributable to primary infection were primarily found in the northeastern region, while secondary infection clusters were mainly in the southeastern region (Figures S8 a and b).* The map (Figure S8 a and b) has also been uploaded.
2. Your observation is correct. Regions are presented in the table, while we used municipalities for the special analysis. Since Mexico has 2469 municipalities, we had to categorize them into states and further into regions to get meaningful estimates for our regression models.
3. Thank you for the suggestion to add the maps for different serotypes. A map showing the distribution of cases by serotypes has been described in a previous publication. Beginning **line 327, page 14,** we have added the statement ‘*A map showing the distribution of DENV serotypes across Mexico [7] has characterized hotspots of DENV-3 in both Northeastern and Southeastern Mexico.*’

DISCUSSION

**Line 289 – In this sentence, it is not clear if the analysis refers to clusters of severe cases of DENV-3 with primary infection, or if they only associate the higher prevalence of DENV-3 in the region with clusters of severe cases of primary infection.**

Response: The statement references table S2. To clarify this, starting from **line 299, page 13** in the results section, the sentence has been modified to *‘Furthermore, bivariate analysis from Table S2 shows that, when compared to all regions, other than the Southeast, DENV-3 was most predominant in the northeast region.’*

SUMMARY AND GENERAL COMMENTS

**Comment: The study addresses a significant issue for the understanding of factors associated with dengue severity, especially age and secondary infection. The methods are adequate to answer the study questions and it was well performed.**

Response: Thank you. We appreciate this feedback.

PDF Review

**General Edits:**

Comments: Thank you for the corrections made in the manuscript. These have been highlighted and accepted in the final document.

**Comments and Questions:**

**Abstract Comment: Does this happen before age? If you're controlling for confounders, they should precede or occur at the same time as "age."**

Response: That is correct. DENV serotype-specific serotype data was collected for the participants at the same time information about age was collected.

**Comment: Need to define this acronym**

Response: Thank you. This has been spelled on **line 74, page 3** as ‘*dengue virus (DENV)*’

**Comment: I may be missing something, but I don't think the 3 serotypes have not been introduced?**

Response: To improve clarity, beginning **line 83, page 3**, the sentence has been modified to ‘*While the IgM disappears in 2-3 months, IgG persists in the body for life, providing lifelong immunity against the infecting serotype, but not any of other the three dengue serotypes [4]*.’

**Comments: It would be helpful to have a few sentences explaining why it is of interest/importance to study these direct and indirect effects. What does this mediation analysis add to the existing literature, and what would it add to dengue research?**

**Based on this description of the statistical analysis, it is still unclear to me how you are estimating the direct and indirect effects of interest. I would suggest making this more explicit here.**

Response: Beginning line **100, page 4**, the paragraph has been modified to include ‘. *Hence, our study aimed to quantify the direct and indirect effect of age on severe dengue through the mediating role of secondary infection (Figure 1). This mediation analysis was performed to help provide information about the mechanism with which age predicts dengue severity. We hypothesized that the effect of age on dengue severity would be insignificant after the introduction of infectivity status into a model predicting severe dengue, after controlling for confounding variables. Further, we hypothesized that the effect of infectivity status would be moderated by DENV serotype.*’

**Comment: In this introduction, I believe there needs to be a setup (e.g., background, rationale) for the spatial clustering analysis mentioned in the abstract**

Response: Beginning **line 106, page 4**, the following sentences ‘*We further characterized the spatial distribution of infectivity status for severe dengue across Mexico. While a secondary infection of dengue may be due to a homologous infection, a secondary infection that is associated with severe dengue is likely due to infection with a heterologous DENV serotype[3]. Hence, we expected to find spatial differences across infectivity status for those with severe dengue. Regional differences of infectivity may require extra vigilance and efforts to control dengue in areas with significant clusters of secondary infection.’* have been added

**Comment: More is needed on the population of interest and how the sample was drawn from the population. For example, what ages did you include? I assume you only used a sample of people who had dengue. How did you identify those people? How did you identify the people who did not have a secondary infection?**

Response: Beginning line **115, page 4**, the sentences ‘*Our population of interest included all individuals diagnosed with dengue in Mexico with information about IgM and IgG serology results from 0 to 100 years. The dataset had information about individuals meeting these criteria from 2012 to 2017*’ have been added.

Classification of the infectivity variable is defined as ‘Mediator variable: A person with IgG positive DF and a current DF diagnosis and IgM positive result were classified as having a secondary infection. Individuals with only IgM positive results were classified as having a primary infection.’, beginning **line 138, page 5**.

**Comment: What are "other classifications"?**

Response: For better clarity, beginning **line 132, page 5**, the sentence has been modified to read ‘Missing *or data entered as ‘other’* were excluded from the analysis.’

**Comment: From the sound of the paper (the title and abstract), your exposure variable is Age only, as you are looking at the direct and indirect effect of Age on DF severity. I'm not sure why these other variables are discussed in the "Exposure variables" section.**

Response: Thank you for this comment. The exposure variable now only includes the age category

**Comment: I would expect a section on "Mediator variables", where you discuss the infectivity status variable.**

Response: Thank you, this section has been added and beginning **line 138, page 5,** it reads ‘*Mediator variable: A person with IgG positive DF and a current DF diagnosis and IgM positive result were classified as having a secondary infection. Individuals with only IgM positive results were classified as having a primary infection.’*

**Comment: At this point, it is still unclear to me what the rationale is for doing a mediation analysis; I'm not sure it helps answer the research question of how infectivity effects on severity change with age.**

**For example, why not do an analysis that looks at the effect of infectivity on dengue severity by age subgroups? In other words, the research question: how does age moderate the effect of infectivity on dengue severity?**

Response: Thank you for reiterating this. Clarifying the aim has been addressed in earlier comments.

**Comment: For this mediation analysis, instead of assessing "the effect of binary mediators and binary outcomes", it sounds instead like you are assessing the 1) direct effect of a categorical age variable on a binary dengue severity variable and 2) indirect effect of a categorical age variable on a binary dengue severity variable through a binary infectivity status variable.**

Response: Thank you for this addition. Beginning **line 161, page 6**, the sentences ‘*The mediation analysis comprised the assessment of 1. the direct effect of a categorical age variable on a binary dengue severity variable and 2) the indirect effect of a categorical age variable on a binary dengue severity variable through a binary infectivity status variable.*’ have been added.

**Comment: Need to briefly introduce the "product method" to show how it estimates the direct and indirect effects of interest**

Response: We appreciate this comment. Beginning **line 164, page 6**, the sentences ‘*Two traditional approaches have been used for mediation analysis in prior literature. A difference method and a product method [8]. When modeling a binary outcome, the estimates from the difference method vary from the product method due to non-collapsibility of the exposure-outcome effect across mediator values [9, 10]. Further, the difference-in-coefficients method conflates the indirect effect when modeling a binary outcome [11]. Hence, the product-of-coefficients was used in our analysis.*’ have been added.

**Comment: No covariates have been introduced yet; I would expect them in the Definition of Variables section.**

Response: Thank you. This has been addressed in previous comments.

**Comment: I would suggest here briefly describing why this is true, i.e., linking the causal effect quantity of interest with the parameters of this regression.**

Response: Beginning **line 181, page 7**, the sentence ‘*Here, X_1_, X_2_ and X_3_ represent ‘>1 to 20’ vs ‘0 to 1’, ‘>20 to 60’ vs ‘0 to 1’ and ‘>60’ vs ‘0-1’ group comparisons.*’ has been added and beginning **line 185, page 7,** the sentence ‘*The relative direct effect of age (x) on disease severity (y) is given as equation 5. In contrast, the relative indirect effect of x on y through infection status (m) is given by the product of the mediation coefficient (b) and age (ᵦ_i_), where i = 1,2,3 (equation 6).*’ has been added.

**Comments: Again, what is (or are) the confounder(s)?**

**Again, what potential confounders?**

Response: These have now been described in under the covariates in the method section beginning **line 141, page 5.**

**Comment: I would put this in the Definition of Variables section above**

Response: Thank you. The definition of age has been previously addressed under the exposure section in methods.

**Comment: What are X1, X2, and X3? Are they the last three age categories? They have not been defined.**

**Again, I think there need to be more explanation/guidance to the reader why this is true and relevant for estimation of the desired direct and indirect effects.**

Response: Thank you. These questions have been addressed in previous comments.

**Comment: Which hierarchical model? I'm not sure the rationale for including this.**

Response: Although a hierarchical model was explored at the beginning, the final model was not. Hence, this has been removed from the manuscript. Beginning **line 198, page 8**, the sentence now reads ‘*Based on the directed acyclic graph (DAG) (Figure 1), several models were explored to determine the direct effect of age on dengue severity, the indirect effect of age moderated by infectivity status on dengue severity and the effect of age on infectivity status.*’

**Comment: Best model for what?**

Response: Beginning **line 201, page 8**, the sentence now reads ‘*Based on the Akaike Information Criterion (AIC) and likelihood ratio test, interactions were explored, and the model without interactions was found to be the best model for the estimation of the relative direct and indirect effects.*’

**Comment: This is the first time this spatial analysis is introduced, and so the reason for including it and the rationale for presenting this procedure are unclear.**

Response: Thank you for the query. The spatial analysis is now first introduced in the introduction section

**Comment: These aren't descriptive statistics as you have inferential results here (eg p-values)**

Response: Thank you for pointing this out. The title of the table now reads ‘Table 2: Age categories stratified by infection status and disease severity’ on **line 239, page 10**.

**Comment: This should go in the Methods section**

Response: Beginning **line 188, page 7**, the formulae for the direct and indirect effects have been moved to the methods section.

**Comment: It would be helpful if, instead of (or in addition to) listing the regression parameter estimates, you present the actual direct, indirect, and total effect estimates. As-is, it's hard to decipher what they are.**

Response: Beginning **line 280, page 12,** the sentence ‘*The total effect for all three group comparisons (X_1_, X_2_ and X_3_) were smaller (12, 13 and 14) than the indirect effects, after summing up the direct and indirect effects. Figure S7 shows* *a summary of the estimates for the direct effect, indirect effect, and the mediation pathway through secondary infection to dengue severity.*’ has been added.

**Comment: Numerical results for this?**

Response: Beginning **line 293, page 13,** the paragraph now reads ‘*Clusters for those with severe dengue attributable to primary infection were primarily found in the northeastern region, while secondary infection clusters were mainly in the southeastern region (Figures S8 a and b). Moran’s I for those with severe dengue who had primary infection was 0.1129. With a critical z-score of 2.72 (p-value = 0.0065), there was less than 1% likelihood that the clustered pattern was due to chance. However, the pattern observed for the clusters who had secondary infection among those with severe dengue did not appear to be significantly different from random (Moran’s I= -0.012, z-score= -0.5351, p-value = 0.5926). The Northeast region had more clusters for severe dengue among those with primary infection.* *Furthermore, bivariate analysis from Table S2 shows that, when compared to all regions, other than the Southeast, DENV-3 was most predominant in the northeast region*.’

**Comment: I'm not sure you can say this unless you have the percentage of the total effect that is due to the indirect effect; I haven't seen that anywhere.**

Response: Beginning **line 303, page 13,** the discussion section now begins with ‘*Individuals between 0-1 year and those 60 years and older have relatively higher odds of severe dengue and need critical management of dengue infection. However, the effect of age on severe dengue appears to be no longer significant after introducing secondary infection status into the causal model estimating the effect of age on severe dengue.*’

**Comment: This was not mentioned in the data collection section**

Response: Beginning **line 157, page 6**, information about missing data has now been presented and it reads ‘*Missing data included 51386 observations that were excluded from the final sample. The final sample size for the analysis was 52,212.*’

Once again, thank you for the helpful and constructive feedback. Thank you for giving us the opportunity to revise and resubmit our manuscript.

Sincerely,

The Authors

1.	Hause AM, Perez-Padilla J, Horiuchi K, Han GS, Hunsperger E, Aiwazian J, et al. Epidemiology of Dengue Among Children Aged < 18 Months-Puerto Rico, 1999-2011. The American journal of tropical medicine and hygiene. 2016;94(2):404-8. Epub 2015/12/30. doi: 10.4269/ajtmh.15-0382. PubMed PMID: 26711519; PubMed Central PMCID: PMCPMC4751950.

2.	Unnikrishnan R, Faizal BP, Vijayakumar P, Paul G, Sharma RN. Clinical and laboratory profile of dengue in the elderly. J Family Med Prim Care. 2015;4(3):369-72. Epub 2015/08/20. doi: 10.4103/2249-4863.161323. PubMed PMID: 26288776; PubMed Central PMCID: PMCPMC4535097.

3.	Soo KM, Khalid B, Ching SM, Chee HY. Meta-Analysis of Dengue Severity during Infection by Different Dengue Virus Serotypes in Primary and Secondary Infections. PLoS One. 2016;11(5):e0154760. Epub 2016/05/24. doi: 10.1371/journal.pone.0154760. PubMed PMID: 27213782; PubMed Central PMCID: PMCPMC4877104.

4.	Khetarpal N, Khanna I. Dengue Fever: Causes, Complications, and Vaccine Strategies. J Immunol Res. 2016;2016:6803098. Epub 2016/08/16. doi: 10.1155/2016/6803098. PubMed PMID: 27525287; PubMed Central PMCID: PMCPMC4971387.

5.	Alahmadi K, Al-Zahrani A. Spatial Autocorrelation of Cancer Incidence in Saudi Arabia. International journal of environmental research and public health. 2013;10:7207-28. doi: 10.3390/ijerph10127207.

6.	Anselin L. Local indicators of spatial association—LISA. Geographical analysis. 1995;27(2):93-115.

7.	Annan E, Bukhari MH, Treviño J, Abad ZSH, Lubinda J, da Silva EAB, et al. The ecological determinants of severe dengue: A Bayesian inferential model. Ecological Informatics. 2023;74:101986. doi: <https://doi.org/10.1016/j.ecoinf.2023.101986>.

8.	VanderWeele TJ. Mediation Analysis: A Practitioner's Guide. Annu Rev Public Health. 2016;37:17-32. Epub 2015/12/15. doi: 10.1146/annurev-publhealth-032315-021402. PubMed PMID: 26653405.

9.	MacKinnon DP, Dwyer JH. Estimating mediated effects in prevention studies. Evaluation review. 1993;17(2):144-58.

10.	Rijnhart JJM, Valente MJ, Smyth HL, MacKinnon DP. Statistical Mediation Analysis for Models with a Binary Mediator and a Binary Outcome: the Differences Between Causal and Traditional Mediation Analysis. Prevention Science. 2021. doi: 10.1007/s11121-021-01308-6.

11.	MacKinnon DP, Lockwood CM, Brown CH, Wang W, Hoffman JM. The intermediate endpoint effect in logistic and probit regression. Clinical Trials. 2007;4(5):499-513.
