# Supplementary material for: Direct and indirect effects of age on dengue severity: The mediating role of secondary infection
Source: PLoS Negl Trop Dis. 2023 Aug 9;17(8):e0011537. doi: 10.1371/journal.pntd.0011537 (PMC10441797; doi:10.1371/journal.pntd.0011537)
Supplement: Attachment — Submitted filename: MS_mediation_analysis_highlights_review.docx [file pntd.0011537.s014.docx]

**Direct and indirect effects of age on dengue severity: the mediating role of secondary infection**

Esther Annan^1^, Jesús Treviño^2^, Bingxin Zhao^3^, Alfonso J. Rodriguez-Morales^4,5,6^, Ubydul Haque^7,8^.

^1^Center for Health and Wellbeing, School of Public and International Affairs, Princeton University, Princeton, NJ, United States

^2^Department of Urban Affairs at the School of Architecture, Universidad Autónoma de Nuevo León, San Nicolás de los Garza, 66455, Nuevo Léon, México

^3^Department of Statistics and Data Science, The Wharton School, University of Pennsylvania, Philadelphia, PA 19104, United States

^4^Grupo de Investigacion Biomedicina, Faculty of Medicine, Fundacion Universitaria Autónoma de las Americas, Pereira, Risaralda, Colombia.

^5^Faculty of Medicine, Institución Universitaria Visión de las Americas, Pereira, Risaralda, Colombia.

^6^Clinical Epidemiology and Biostatistics, Universidad Cientifica del Sur, Lima, Peru.

^7^Rutgers Global Health Institute, New Brunswick, NJ

^8^Department of Biostatistics and Epidemiology, School of Public Health, Rutgers University, Piscataway, NJ, USA

**Keywords:** dengue fever; severe dengue; secondary infection; mediation; serotype; direct and indirect effect; Mexico

*Corresponding Author

E-mail: esther.annan@princeton.edu

**Abstract**

Severe dengue occurrence has been attributed to increasing age and different DENV serotypes that cause secondary infections and immune-enhancing phenomena. Therefore, we examined if the effect of age on dengue severity was mediated by infectivity status while controlling for sex and DENV-specific serotypes. Further, we assessed the spatial clustering of dengue severity for individuals with primary and secondary infection across Mexican municipalities. Health data from 2012 to 2017 was retrieved from Mexico's Ministry of Health. A mediation analysis was performed using multiple logistic regression models based on a directed acyclic graph. The models were explored for the direct effect of age on dengue severity and its indirect impact through secondary infection. In addition, severe dengue clusters were determined in some Northeastern and Southeastern municipalities through spatial analysis. Individuals between the ‘>1-20 years’ [0.45, (CI: 0.37, 0.55)], ‘>20-60 years’ [0.46, (CI: 0.38, 0.57)] and ‘>60 years’ [0.63, (CI: 0.50, 0.80)] had lower odds of severe dengue, when compared with those in the ‘0-1 year’ age group. The effect of age on dengue severity was no longer significant after introducing infectivity status into the logistic regression model, indicating age effect was largely mediated by secondary infection. Clustering of severe dengue among individuals with primary infection in the Northeastern region may point to the high prevalence of DENV-3 in the region. Public health efforts may prevent secondary infection among infants and the aged. In addition, there should be a further investigation into the effect of DENV-3 in individuals with primary disease.

**Author Summary**

Age has been previously found to be associated with severe dengue. However, it is unclear whether this effect is purely due to aging or the predisposition of older individuals to be exposed to secondary infection. Since secondary infection with a dengue serotype other than the infecting serotype during the first infection increases the risk of severe dengue, we explored how much this effect contributed to the effect of age on severe dengue. We performed a mediation analysis and assessed the spatial distribution pattern of primary and secondary infection across Mexico to determine regions vulnerable to severe dengue occurrence. The youngest and oldest population groups had relatively higher odds of severe dengue. However, the effect of age on severe dengue was largely mediated by secondary infection. The spatial analysis also revealed aggregation of severe dengue cases among individuals with primary infection in the Northeastern region, which may be associated with the high prevalence of DENV-3 in the region. Focusing on the prevention of secondary infection among the elderly and infants may help to avert the number of severe dengue cases.

**Introduction**

Dengue fever has in recent years expanded in geographic distribution, causing endemic diseases with seasonal fluctuations across the globe [1-4]. In Mexico, the force of dengue infection between 2008 and 2014 was about 7.1% (5.1%–9.8%), indicating a high endemic transmission [5]. Infection with dengue presents on a spectrum of mild to severe, with economic impact primarily attributable to indirect costs from loss of productivity [6]. The seasonal transmission of dengue may be explained by environmental factors [7], viral evolution, and population-specific immunity against different dengue virus (DENV) serotypes [8]. Individual-level predictors of disease severity have been found to include age, comorbidities, sex, and infection with multiple serotypes [9-14].

After a first epidemic, subsequent epidemics result in fewer cases due to herd immunity, and younger individuals become progressively susceptible as the immune population ages [15]. As epidemics continue over time, the age-specific seroprevalence is expected to change, with small outbreaks involving younger generations [15]. The risk of having a classical dengue disease, which traditionally reflects less severe symptoms, has been found to increase with age after a person experiences primary infection [16].

Primary dengue infection is characterized by high titers of immunoglobulin M (IgM) in 3-5 days and immunoglobulin G (IgG) antibodies from the sixth to the tenth day after symptom onset [17]. While the IgM disappears in 2-3 months, IgG persists in the body for life, providing lifelong immunity against the infecting serotype, but not any of other the three dengue serotypes [17]. The same serotype as the first infection, or any of the other three serotypes, may cause secondary dengue infections. When a person is re-infected with a different or unencountered DENV serotype, this usually leads to classical dengue fever (DF) or dengue without warning signs [17]. Of the people with a secondary infection, 2% - 3% progress to dengue with warning signs, which may further result in severe dengue and death [17]. DHF is characterized by hemorrhagic signs like thrombocytopenia, petechiae and epistaxis, while DSS occurs due to leakage of intravascular fluids and proteins into perivascular spaces [18]. Antibody-dependent enhancement (ADE) occurs because of secondary infection with a heterologous DF serotype and is associated with severer infections. However, not all severe infections are a result of a secondary infection [17].

An increase in an individual's age increases the likelihood of exposure to a secondary infection, and the outcome of dengue serotype 2 (DENV-2) secondary infection has been found to predict the risk of severe clinical presentation of DF [19]. The association between secondary serologic response and severe dengue has been established in the previous literature [20], and prior studies have explored the influence of age on clinical dengue presentation. Age has been found to be associated with an increased risk of clinical dengue [1]. However, to the best of our knowledge, the mediation effect of secondary infection in the causal pathway between age and severe dengue has not been studied. Hence, our study aimed to quantify the direct and indirect effect of age on severe dengue through the mediating role of secondary infection (Figure 1). This mediation analysis was performed to help provide information about the mechanism with which age predicts dengue severity. We hypothesized that the effect of age on dengue severity would be insignificant after the introduction of infectivity status into a model predicting severe dengue, after controlling for confounding variables. Further, we hypothesized that the effect of infectivity status would be moderated by DENV serotype. We further characterized the spatial distribution of infectivity status for severe dengue across Mexico. While a secondary infection of dengue may be due to a homologous infection, a secondary infection that is associated with severe dengue is likely due to infection with a heterologous DENV serotype [21]. Hence, we expected to find spatial differences across infectivity status for those with severe dengue. Regional differences of infectivity may require extra vigilance and efforts to control dengue in areas with significant clusters of secondary infection.

**Methods**

***Data Collection and Preprocessing***

Data used for the analysis was acquired from the Ministry of Health, Mexico. Our population of interest included all individuals diagnosed with dengue in Mexico with information about IgM and IgG serology results from 0 to 100 years. The dataset had information about individuals meeting these criteria from 2012 to 2017. Hence, data for all variables of interest were compiled from 2012 to 2017, and a cross-sectional study was performed. Hence, the analysis was restricted to observations from these six years. The Ministry of Health data reports non-identifiable clinical findings from notifiable health units in 2,469 Mexican municipalities. This study was approved as an exempt category by the North Texas Regional Institutional Review Board and the Universidad Autónoma De Nuevo León.

***Definition of Variables***

Outcome variable: Individuals with signs of clinical dengue were further tested for laboratory-confirmed evidence of non-structural protein (NS1) of DENV. An individual with positive NS1 results was classified as having dengue. DF is classified in the dataset as non-severe dengue, severe dengue, and dengue with alarming signs. These classifications are closely synonymous with DF, dengue hemorrhagic fever (DHF), and dengue shock syndrome (DSS), respectively [22]. DF severity was categorized as non-severe dengue and severe dengue. Based on the world health organization’s emphasis on critical care for individuals with alarming signs, people with serious dengue or dengue with alarming signs had severe dengue, while those with non-serious dengue were in the ‘non-severe dengue’ group [23]. Missing data or data entered as ‘other’ were excluded from the analysis.

Exposure variable: Age was categorized into a four-level variable due to an established bimodal age pattern among individuals with DENV-2 secondary infection[19]. Age (in years) was categorized as '0-1','> 1-20','> 20-60', '>60' to account for the infant and elderly populations that have a higher risk for severe dengue [24, 25].

Mediator variable: A person with IgG positive DF and a current DF diagnosis and IgM positive result were classified as having a secondary infection. Individuals with only IgM positive results were classified as having only a primary infection and no secondary infection.

Covariates: Infecting DENV serotypes were classified based on polymerase chain reaction RT-qPCR results [26] and was reported in the dataset as DENV-1, DENV- 2, DENV-3, or DENV-4. Demographic variables reported in the dataset include age and sex. Sex was categorized as male or female. Baja California, Baja California Sur, Sonora, Sinaloa, and Chihuahua were defined as the 'Northwest' region. Durango, Coahuila, Nuevo Leon, and Tamaulipas were defined as ‘Northeast’ region. Zacatecas, San Luis Potosi, San Luis Potosi, Aguascalientes, Guanajuato, Queretaro, Nayarit, Jalisco, Colima, and Michoacan were categorized as ‘Center west’ region. Hidalgo, Mexico City, Distrito Federal, Morelos, Puebla, Tlaxcala were in the ‘Center’ region, while Oaxaca, Guerrero, Veracruz, Chiapas, Tabasco, Campeche, Quintana Roo and Yucatan were classified under ‘Southeast’ region.

***Statistical Analysis***

Dengue severity was tabulated by demographic and predictor variables. Variances across subgroups were tested for statistical significance using chi-square tests for categorical variables and independent sample t-tests for continuous variables. The Type I error alpha value was set at 0.05 for all significance tests. Univariate analyses were conducted for descriptive purposes, and bivariate analyses were performed to determine associations between different predictors and dengue severity. All bivariate analyses were adjusted for False Discovery Rate (FDR). Missing data included 51386 observations that were excluded from the final sample. The final sample size for the analysis was 52,212.

*Mediation Analysis*

The mediation analysis comprised the assessment of 1) the direct effect of a categorical age variable on a binary dengue severity variable and 2) the indirect effect of a categorical age variable on a binary dengue severity variable through a binary infectivity status variable. A regression-based approach may be used in mediation analysis to assess the effect of binary mediators on binary outcomes. Two traditional generalized linear regression-based approaches have been used for mediation analysis in prior literature: a difference method and a product method [27]. When modeling a binary outcome, the estimates from the difference method vary from the product method due to non-collapsibility of the exposure-outcome effect across mediator values [28, 29]. Further, the difference-in-coefficients method conflates the indirect effect when modeling a binary outcome [30]. Hence, the product-of-coefficients was used in our analysis. When using the product method in the mediation analysis, the procedure comprises the estimation of two regression equations; equation 1 evaluates the effect of the exposure (x) and covariate (c) on the mediator (m), while equation 2 estimates the effect of x, m and c on the outcome (Y) [27].

logit {P (M=1|a, c)} = ᵦ_o_ + ᵦ_1_ x+ ᵦ׳_2_ c.………………………….…………. (1)

logit {P (Y =1|x, m, c)} = θ_o_ + θ_1_ x + θ_2_ m + θ_3_c …………………………...…..... (2)

where, respectively, ᵦ_0_ + ᵦ_1_ and ᵦ׳ represent the intercept and effects of age (x) and confounder (c) on infectivity status. Similarly, θ_0_, θ_1_, θ_2_, and θ_3_ represent the intercept and respective effects of age (x), infectivity status (m) and confounder (c) on disease severity classification.

In their paper, Hayes and Preacher introduced the concept of relative indirect and direct total effects as multi-categorical independent variables [31]. To compute this, the independent variable 'age_cat' was dummy coded, with the '0-1' group as the reference. Equations (1) and (2) were therefore transformed into (3) and (4), respectively. Here, X_1_, X_2_ and X_3_ represent ‘>1 to 20’ vs ‘0 to 1’, ‘>20 to 60’ vs ‘0 to 1’ and ‘>60’ vs ‘0-1’ group comparisons. This may also be visualized in the path diagram (Figure 2).

logit {P (M=1|a, c)} = ᵦ_o_ + ᵦ_1_ x_1_ + ᵦ_2_x_2_ + ᵦ_3_ x_3_+ ᵦ׳_4_ c.…….…………. (3)

logit {P (Y =1|x, m, c)} = θ_o_ + θ_1_ x_1_ + θ_2_ x_2_ + θ_3_ x_3_ + *b*m + θ׳_4_c _…_…...…....…. (4)

The relative direct effect of age (x) on disease severity (y) is given as equation 5. In contrast, the relative indirect effect of x on y through infection status (m) is given by the product of the mediation coefficient (b) and age (ᵦ*_i_*), where *i* = 1,2,3 (equation 6).

Relative Direct Effect = θ*_i_ ……….…………………………………………………* (5)

Relative Indirect Effect *=* ᵦ*_i_* b ………………………………………………. (6)

The word 'relative' is used to emphasize that the effects are relative to the reference group '0-1' and would change if the reference group were substituted [31]. Since the independent variable has multiple categories, the total effect was estimated by the parameter estimates from the dummy coded groups and given by equation (7). The estimates produced by the mean differences between the age groups are referred to as relative total effects. The total relative effect may also be calculated by summing up the relative direct and indirect effects (8).

logit {P (Y =1|x, c)} = ф_o_ + ф_1_ x_1_ + ф_2_ x_2_ + ф_3_ x_3_ + ф׳_4_c…...…...…. (7)

ф*_i_* _=_ θ*_i_* + ᵦ*_i_ b* …………………………. (8)

Based on the directed acyclic graph (DAG) (Figure 1), several models were explored to determine the direct effect of age on dengue severity, the indirect effect of age mediated by infectivity status on dengue severity and the effect of age on infectivity status. For each model, sex, region, diabetes, hypertension and DENV serotype were used as covariates. Based on the Akaike Information Criterion (AIC) and likelihood ratio test, interactions were explored, and the model without interactions was found to be the best model for the estimation of the relative direct and indirect effects.

*Spatial Analysis*

Severe and non-severe dengue cases were summed up by secondary infection status. That was used in a cluster analysis where the spatial variability of severe dengue was compared by infection status across Mexican municipalities. The spatial distribution of severe and non-severe dengue was visualized for both primary and secondary infection. An optimized outlier analysis of severe dengue was then performed for each infection status. Statistically significant spatial clusters of high values (hot spots) and low values (cold spots) were identified using the Anselin Local Moran's I (LMi Index) statistic. The Anselin local Moran’s I is a local indicator of spatial association (LISA). The LISA for an observation shows the extent to which there is significant spatial clustering of similar values around that observation, while the sum of all LISAs correlated with the global spatial association [32]. The statistics behind the Anselin formula is further explained elsewhere [33]. The FDR correction method was used at a 5% error rate to correct for multiple testing and spatial dependence [34].

*Coding and Environment*

Preprocessing of data, analysis, and generation of figures were done using SAS (version 9.4, SAS Institute Inc., Cary, NC, USA).

**Results**

Of the 52,212 individuals with reported data, 60% were female, with roughly half (50%) living in the Southeast region and 81.2% having non-severe dengue (Table 1). The average age was 32 years, ranging from 0 to 100 years, with females being averagely older (33 years) than males (30 years). The difference between primary and secondary infection by gender was not statically significant (ꭓ^2^ = 0.007, p= 0.9447 (Table S1). On average, individuals with secondary infection were older than those with primary infection (Figure S1). This difference was statistically significant [t (_7961.7_) = -15.99, p < 0.0001]. The prevalence of secondary infection was higher among individuals with severe dengue (95%) than those with non-severe dengue (87%). Descriptive statistics for the age category are in Table 2. A chi-square assessment revealed statistically significant differences between age categories for infection status (ꭓ^2^= 211.4, <0.0001) and disease severity (ꭓ^2^= 178.0, < 0.0001). Increasing age was associated with higher proportions of secondary infection, while the extreme age categories (youngest and oldest) were significantly associated with higher proportions of disease severity.

Table 1: Demographic characteristics by dengue classification

| Parameter | Dengue Classification, n (%) | | | | |
| --- | --- | --- | --- | --- | --- |
|  |  | Severe Dengue | | Non-Severe Dengue | All |
| **Sex | Male | 4645 (31.42%) | | 16243 (77.76%) | 20888 (40.01%) |
|  | Female | 5181 (16.54%) | | 26143 (83.46%) | 31324 (59.99%) |
| **Region | Center | | 479 (4.87%) | 2156 (5.09%) | 2635 (5.05%) |
|  | Center- West | | 966 (9.83%) | 6855 (16.17%) | 7821 (14.98%) |
|  | North - East | | 1173 (11.94%) | 11100 (26.19%) | 12273 (23.51%) |
|  | North - West | | 435 (4.43%) | 2837(6.69%) | 3272 (6.27%) |
|  | South - East | | 6773 (68.93%) | 19438 (45.86%) | 26211 (50.20%) |
| ^NS^ Age in years (SD) |  | 32 (±19.80) | | 32 (±18.71) | 32 (±18.92) |
| Total (n) |  | 9826 (18.18%) | | 42386 (81.18%) | 52212 (100%) |

** Chi-square performed for group differences had p <0.001

^NS^ P-value of T-test not significant

Table 2: Age categories stratified by infection status and disease severity

|  | Infection Status | | Chi-square (p-value) | Disease Severity | | Chi-square (p-value) |
| --- | --- | --- | --- | --- | --- | --- |
| Age Category (years) | Secondary Infection | Primary Infection | ꭓ^2^= 273.0, <0.0001 | Severe Dengue | Non-Severe Dengue | ꭓ^2^= 68.3, < 0.0001 |
| 0- 1 | 443 (75.3%) | 145 (24.7%) |  | 125 (21.3%) | 463 (78.7%) |  |
| >1 – 20 | 14319 (86.0%) | 2325 (14.0%) |  | 3287 (19.8%) | 13357 (80.2%) |  |
| >20 – 60 | 26913 (89.0%) | 3316 (11.0%) |  | 5363 (17.7%) | 24866 (82.3%) |  |
| >60 | 4393 (92.5%) | 357 (7.5%) |  | 1050 (22.1%) | 3700 (77.9%) |  |

Figures S2 and S3 show the proportions of severe (a) and non-severe (b) dengue and the proportions of infection status for each category. Hence, all four proportions add up to 100% for every age Compared to the other ages, there was a lower frequency of secondary infection among individuals about 0-5 years for both severe and non-severe dengue (Figure S2a&b). The average frequency of dengue among those with secondary infection from about five years to 60 years was about 20% and 70%, respectively, for those with severe and non-severe dengue. However, above 60 years, there was a steeper increase in the frequency among those with severe dengue than those with non-severe dengue (Figure S2a&b). The frequency of non-severe dengue decreased with age among those with primary infection. However, the frequency of severe dengue among those with primary infection and severe dengue remained around 5% for the entire age distribution except for those above 60 years who had higher frequencies of severe dengue (Figure S2a&b). Differences observed between primary and secondary infection for severe dengue (t-value= -2.56, p=0.001) was significant but not for non-severe dengue (t-value=-0.99, p=0.3219). Figures S3a &b show variations in the frequency of primary and secondary infection from 2012 to 2017 for severe and non-severe dengue. Cochrane-Armitage trend test for the difference between primary and secondary infection for severe dengue (Z = 2.01, p= 0.04) and non-severe dengue (Z= -14.67, p<0.0001) were both statistically significant. For those with primary infection, there is an observed increase in the proportion of severe and non-severe dengue between 2016 and 2017. Although secondary infection was commonest in the Southeast region (55.8%), primary infection was commonest in the Northeast region (80.5%) (Table S1 & Figure S4). Regional differences were statistically significant [ꭓ^2^ = 12775.3, p< 0.0001].

DENV-2 was the commonest serotype among individuals with severe and non-severe dengue (Table 3). The serotype distribution pattern was similar for both sexes (Figure S5). However, the proportion of those with secondary infection who had severe dengue was higher than those with non-severe dengue. Observed serotype and infection status differences were statistically significant (Table 3). The Southeast region had the highest proportion of DENV-2, DENV-3, and DENV-4 and the second highest for DENV-1 (Table S2). Apart from the Southeast region, the Northeast had the highest proportion of DENV-3 and a higher proportion of DENV-4. In contrast, the Center west region had higher proportions of DENV-1 and DENV-2 than the other regions.

Table 3: Proportions and p-value for different chi-squared test results with 95% significance

| Parameter | | Dengue Classification (%) | | |
| --- | --- | --- | --- | --- |
|  |  | Severe Dengue | Non-Severe Dengue | Chi-square p-value |
| Serotype | DENV- 1 | 58 (0.59%) | 233 (0.55%) | <.0001 |
|  | DENV- 2 | 183 (1.86%) | 289 (0.68%) |  |
|  | DENV- 3 | 5 (0.05%) | 18 (0.04%) |  |
|  | DENV- 4 | 3 (0.03%) | 37 (0.09%) |  |
|  | Missing | 9577 (97.47%) | 41809 (98.64%) |  |
| Secondary infection | Yes | 9333 (94.98%) | 36735 (86.67%) | <.0001 |
|  | No | 493 (5.02%) | 5651 (13.33%) |  |

Compared to those within the '0-1 year’ age range, individuals within the '>1-20 years' [0.45, (CI: 0.37, 0.55)], '> 20-60 years' [0.46, (CI: 0.38, 0.57)] and '>60 years’ age group [0.63, (CI: 0.50, 0.80)] had lower odds of severe dengue. The effect of age on dengue severity was no longer significant after introducing infectivity status into the logistic regression model (Table 4). The relative indirect effects of the age categories on disease severity are shown in Figure S6 and in equations (9), (10), and (11).

ᵦ_1 *_ *_b_* _=_ 0.7336 x 1.412 = 1.036 ………………………………………. (9)

ᵦ_2 *_ *_b_* _=_ 1.8606 x 1.412 = 2.627 ………………………………………. (10)

ᵦ_3 *_ *_b_* _=_ 2.5057 x 1.412 = 3.538 ………………………………………. (11)

The total effect for all three group comparisons (X_1_, X_2_ and X_3_) were smaller (12, 13 and 14) than the indirect effects, after summing up the direct and indirect effects. Figure S7 shows a summary of the estimates for the direct effect, indirect effect, and the mediation pathway through secondary infection to dengue severity.

Table 4: Estimated coefficients from equations (3) – (5) controlled for hypertension, diabetes, region and DENV serotype, and estimated in SAS using indicator coding with statistical errors in parenthesis.

| Outcome | M | |  |  |  | Y | | | |  |
| --- | --- | --- | --- | --- | --- | --- | --- | --- | --- | --- |
|  |  | Coefficient (SE) | OR [CI] |  | Coefficient (SE) | | OR [CI] |  | Coefficient (SE) | OR [CI] |
| Indicator coding |  |  |  |  |  | |  |  |  |  |
| Constant | ᵦ_o_ | -1.091 (1.15) |  | ф_o_ | 0.114 (0.104) | |  | θ_o_ | -0.898 (0.970) ^NS^ |  |
| ’>1-20’ *vs* ‘0-1’ **(X_1_)** | ᵦ_1_ | 0.734 (1.14) | 2.08 [0.22, 19.63] | ф_1_ | -0.795 (0.103) * | | 0.45 [0.37, 0.55] | θ_1_ | -0.510 (0.957) **^NS^** | 0.94 [0.64, 1.38] |
| ’>20-60’ *vs* ‘0-1’ **(X_2_)** | ᵦ_2_ | 1.861 (0.14) | 6.43 [0.68, 60.47] | ф_2_ | -0.769 (0.103) * | | 0.46 [0.38, 0.57] | θ_2_ | -0.537 **(**0.958**) ^NS^** | 0.85 [0.54, 1.34] |
| ’>60 years’ *vs* ‘0-1’ **(X_3_)** | ᵦ_3_ | 2.506 (1.19) * | 12.25 [1.19, 125.87] | ф_3_ | -0.459 (0.121) * | | 0.63 [0.50, 0.80] | θ_3_ | -0.753 (1.012) **^NS^** | 0.93 [0.47, 1.84] |
| Infection status **(M)** |  |  |  |  |  | |  | *b* | 1.412 (0.182) * | 3.13 [2.19, 4.74] |

^+^ P-value of significance test < 0.0001

^NS^ P-value not significant

Total effect (X_1_) = 1.036 – 0.510 = 0.53 …………………………………………. (12)

Total effect (X_2_) = 2.627 - 0.537 = 2.09 …………………………………………. (13)

Total effect (X_3_) = 3.538 - 0.753 = 2.78 …………………………………………. (14)

Clusters for those with severe dengue attributable to primary infection were primarily found in the northeastern region, while secondary infection clusters were mainly in the southeastern region (Figures S8 a and b). Moran’s I for those with severe dengue who had primary infection was 0.1129. With a critical z-score of 2.72 (p-value = 0.0065), there was less than 1% likelihood that the clustered pattern was due to chance. However, the pattern observed for the clusters who had secondary infection among those with severe dengue did not appear to be significantly different from random (Moran’s I= -0.012, z-score= -0.5351, p-value = 0.5926). The Northeast region had more clusters for severe dengue among those with primary infection. Furthermore, bivariate analysis from Table S2 shows that, when compared to all regions, other than the Southeast, DENV-3 was most predominant in the northeast region.

**Discussion**

Individuals between 0-1 year and those 60 years and older have relatively higher odds of severe dengue and need critical management of dengue infection. However, the effect of age on severe dengue appears to be no longer significant after introducing secondary infection status into the causal model estimating the effect of age on severe dengue. This suggests that assessing an individual's risk for secondary infection may be more helpful in dengue prevention initiatives. Two things common to the Northeastern region between 2012 and 2017 were the relatively higher prevalence of DENV-3 compared to other serotypes and the presence of severe dengue clusters among individuals with primary infection. While efforts must be taken to halt the secondary transmission of DF, rigorous measures in areas newer to DF spread must be considered to address DF complications. Surveillance among dengue-naïve populations should pay particular attention to the introduction of DENV-3 serotypes.

Children below 14 years and adults older than 50 years have been found to have a higher death and hospitalization rate [19]. A similar trend was seen in our study population among individuals with secondary infection, where the frequency of dengue severity slightly decreases from about five years and increases again from 60. The association between higher age and secondary infection is consistent with the literature [35]. Furthermore, the association between severe dengue and ageing populations [35] may be explained by the higher propensity of exposure to a second infection as a person ages. When an individual develops ADE from initial infection [36], cross-reaction with the second infecting virus results in immune complexes that result in cytokine release later in the infection process, further leading to vascular permeability, shock, and increasing the possibility of death [36].

Characterization of DENV-2 in the Southeastern region has been associated with the American/Asian genotypes from Southeast Asia [37]. Since the Southeastern region had the highest proportion of DENV-2, DENV-3, and DENV-4 serotypes, the association between the southeastern region and dengue severity clusters among those with secondary infection may be attributable to the prevalence of old circulating genotypes of the DENV-2, DENV-3, and DENV-4 serotypes in the region.

Apart from the Southeastern region, DENV-3 was also prevalent in the Northeastern region. A map showing the distribution of DENV serotypes across Mexico [38] has characterized hotspots of DENV-3 in both Northeastern and Southeastern Mexico. The association between the northeastern region and severe dengue among those with primary infection suggests a possible link between the predominantly circulating serotype and disease severity. DENV-3 has been associated with severe dengue for individuals with primary infection, while secondary infection with DENV-1, DENV-2, and DENV-4 are primarily associated with severe dengue [21]. Furthermore, the significant clustering of dengue severity among those with primary infection in the Northwestern region may be related to the migration of DENV serotypes towards the north as climate change may favor *Aedes aegypti* dissemination towards central and northern regions [39].

The association between increasing age and dengue severity among individuals with both primary and secondary infection has been established [1]. However, in our study, this was particularly true for individuals older than 60. After controlling for sex, serotype, region, hypertension, diabetes and age, individuals with secondary infection had 3.1 [CI: (2.2, 4.7)] times the odds of having severe dengue. Furthermore, after adding infectivity status into the model, the relationship between age and disease severity was no longer significant. This study shows that, rather than a linear relationship, extreme age groups are at an increased risk of severe dengue and this relationship is largely mediated by infectivity status. While age would indicate more years at risk and an increased likelihood of severe infection, it has been suggested that infants within 6-12 months may be at risk of severe dengue due to placental transfer of maternal IgG [40]. Furthermore, the opposite directions of the direct and indirect effects indicate that there is inconsistent mediation [41]. Hence, the effect of age on severe dengue is due to an indirect mechanism through the infectivity status.

This study has several limitations. First, some studies have linked the history of two or more previous infections with a decreased risk of a new infection [8]. This hypothesis could not be tested in our population since information about several prior dengue infections in the past was unavailable. Secondly, there was a vast amount of missingness, particularly for the serotype data. However, the final sample size was 52,212, and 827 serotype records were available for analysis, with 9577 missing severe dengue (97.47%) classification and 41809 missing non-severe dengue (98.64%) classification (Table 3). Missingness was assumed to be random since the proportion of missingness across disease severity and covariates were proportional, and a complete case analysis was performed for all regression models.

**Conclusion**

Understanding the causal pathways and mechanisms with which age affects dengue severity allows targeting the right population group for interventions. The mediation effect of secondary infection status on dengue severity will enable scientists to focus on ways to prevent dengue re-infection and active management of patients with prior history of dengue, as well as in dengue-naïve populations. Primary infection in Northeastern Mexico and secondary infection in other regions of Mexico require continued surveillance. Furthermore, through the indirect effect of secondary infection, the aged population are at an increased risk of dengue fever complications and requires critical care and management.

**Figure legends**

Figure 1. Directed Acyclic Graph (DAG) of factors associated with dengue severity

Figure 2: Path Diagram showing indicator coding

Figure S1: Age distribution for different infection status categories

Figure S2a: Frequency polygon of Age pattern for severe dengue

Figure S2b: Frequency polygon of Age pattern for non-severe dengue

Figure S3a Trend of infection status from 2012 to 2017 for severe dengue

Figure S3b Trend of infection status from 2012 to 2017 for non-severe dengue

Figure S4: Plot of infection status by dengue severity for each region

Figure S5. Graph of serotype distribution by sex and dengue severity

Figure S6: Path Diagram showing regression coefficients

Figure S7: Effect estimates showing the direct effect, and indirect effect of age, and the mediation effect of secondary infection on dengue severity. On the left is the direct effect of age on dengue severity. In the middle is the indirect effect of age on dengue severity, derived from mediation pathway estimates on the right. Here, a= ‘>1-20’ vs ‘0-1’, b=’>20-60’ vs ‘0-1’, and c=’>60’ vs ‘0-1’.

Figure S8a: Severe Dengue Hotspot among individuals with primary infection

Figure S8b: Severe Dengue Hotspot among individuals with secondary infection

Table S1: Demographic characteristics by infection classification

Table S2: Relationship between the region and serotype distribution.

**References.**

1.	Thai KT, Nishiura H, Hoang PL, Tran NT, Phan GT, Le HQ, et al. Age-specificity of clinical dengue during primary and secondary infections. PLoS Negl Trop Dis. 2011;5(6):e1180. Epub 2011/06/30. doi: 10.1371/journal.pntd.0001180. PubMed PMID: 21713018; PubMed Central PMCID: PMCPMC3119638.

2.	Gibbons RV, Vaughn DW. Dengue: an escalating problem. Bmj. 2002;324(7353):1563-6.

3.	Thai KT, Cazelles B, Nguyen NV, Vo LT, Boni MF, Farrar J, et al. Dengue dynamics in Binh Thuan province, southern Vietnam: periodicity, synchronicity and climate variability. PLoS neglected tropical diseases. 2010;4(7):e747.

4.	Musso D, Rodriguez-Morales AJ, Levi JE, Cao-Lormeau VM, Gubler DJ. Unexpected outbreaks of arbovirus infections: lessons learned from the Pacific and tropical America. Lancet Infect Dis. 2018;18(11):e355-e61. Epub 2018/06/24. doi: 10.1016/s1473-3099(18)30269-x. PubMed PMID: 29934112.

5.	Nealon J, Bouckenooghe A, Cortes M, Coudeville L, Frago C, Macina D, et al. Dengue Endemicity, Force of Infection, and Variation in Transmission Intensity in 13 Endemic Countries. The Journal of Infectious Diseases. 2020;225(1):75-83. doi: 10.1093/infdis/jiaa132.

6.	Laserna A, Barahona-Correa J, Baquero L, Castañeda-Cardona C, Rosselli D. Economic impact of dengue fever in Latin America and the Caribbean: a systematic review. Rev Panam Salud Publica. 2018;42:e111. Epub 2019/05/17. doi: 10.26633/rpsp.2018.111. PubMed PMID: 31093139; PubMed Central PMCID: PMCPMC6386068.

7.	Herrera-Martinez AD, Rodríguez-Morales AJ. Potential influence of climate variability on dengue incidence registered in a western pediatric Hospital of Venezuela. Trop Biomed. 2010;27(2):280-6. Epub 2010/10/22. PubMed PMID: 20962726.

8.	Amaya-Larios IY, Martínez-Vega RA, Diaz-Quijano FA, Sarti E, Puentes-Rosas E, Chihu L, et al. Risk of dengue virus infection according to serostatus in individuals from dengue endemic areas of Mexico. Scientific Reports. 2020;10(1):19017. doi: 10.1038/s41598-020-75891-z.

9.	Kyle JL, Harris E. Global spread and persistence of dengue. Annu Rev Microbiol. 2008;62:71-92.

10.	Lee I-K, Hsieh C-J, Lee C-T, Liu J-W. Diabetic patients suffering dengue are at risk for development of dengue shock syndrome/severe dengue: Emphasizing the impacts of co-existing comorbidity(ies) and glycemic control on dengue severity. Journal of Microbiology, Immunology and Infection. 2020;53(1):69-78. doi: <https://doi.org/10.1016/j.jmii.2017.12.005>.

11.	Nunes PC, Sampaio SA, da Costa NR, de Mendonça MC, Lima Mda R, Araujo SE, et al. Dengue severity associated with age and a new lineage of dengue virus-type 2 during an outbreak in Rio De Janeiro, Brazil. J Med Virol. 2016;88(7):1130-6. Epub 2016/04/12. doi: 10.1002/jmv.24464. PubMed PMID: 27061403.

12.	Pongpan S, Wisitwong A, Tawichasri C, Patumanond J, Namwongprom S. Development of dengue infection severity score. ISRN Pediatr. 2013;2013:845876-. doi: 10.1155/2013/845876. PubMed PMID: 24324896.

13.	Anders KL, Nguyet NM, Chau NV, Hung NT, Thuy TT, Lien le B, et al. Epidemiological factors associated with dengue shock syndrome and mortality in hospitalized dengue patients in Ho Chi Minh City, Vietnam. The American journal of tropical medicine and hygiene. 2011;84(1):127-34. Epub 2011/01/08. doi: 10.4269/ajtmh.2011.10-0476. PubMed PMID: 21212214; PubMed Central PMCID: PMCPMC3005500.

14.	Xu C, Pang J, Hsu JP, Leo YS, Lye DCB. Differences in clinical features and dengue severity between local and migrant Chinese with dengue infection in Singapore. PLoS One. 2018;13(8):e0201441. Epub 2018/08/16. doi: 10.1371/journal.pone.0201441. PubMed PMID: 30110348; PubMed Central PMCID: PMCPMC6093606.

15.	Focks D, Barrera R. Dengue Transmission Dynamics: Assessment And Implications For Control. WHO Report of the Scientific Working Group meeting on Dengue, Geneva. 2006.

16.	Egger JR, Coleman PG. Age and clinical dengue illness. Emerging infectious diseases. 2007;13(6):924-5. doi: 10.3201/eid1306.070008. PubMed PMID: 17553238.

17.	Khetarpal N, Khanna I. Dengue Fever: Causes, Complications, and Vaccine Strategies. J Immunol Res. 2016;2016:6803098. Epub 2016/08/16. doi: 10.1155/2016/6803098. PubMed PMID: 27525287; PubMed Central PMCID: PMCPMC4971387.

18.	Morens DM, Fauci AS. Dengue and Hemorrhagic FeverA Potential Threat to Public Health in the United States. JAMA. 2008;299(2):214-6. doi: 10.1001/jama.2007.31-a.

19.	Guzmán MG, Kouri G, Bravo J, Valdes L, Susana V, Halstead SB. Effect of age on outcome of secondary dengue 2 infections. International Journal of Infectious Diseases. 2002;6(2):118-24. doi: <https://doi.org/10.1016/S1201-9712(02)90072-X>.

20.	Fried JR, Gibbons RV, Kalayanarooj S, Thomas SJ, Srikiatkhachorn A, Yoon IK, et al. Serotype-specific differences in the risk of dengue hemorrhagic fever: an analysis of data collected in Bangkok, Thailand from 1994 to 2006. PLoS Negl Trop Dis. 2010;4(3):e617. Epub 2010/03/09. doi: 10.1371/journal.pntd.0000617. PubMed PMID: 20209155; PubMed Central PMCID: PMCPMC2830471.

21.	Soo KM, Khalid B, Ching SM, Chee HY. Meta-Analysis of Dengue Severity during Infection by Different Dengue Virus Serotypes in Primary and Secondary Infections. PLoS One. 2016;11(5):e0154760. Epub 2016/05/24. doi: 10.1371/journal.pone.0154760. PubMed PMID: 27213782; PubMed Central PMCID: PMCPMC4877104.

22.	Phanitchat T, Zhao B, Haque U, Pientong C, Ekalaksananan T, Aromseree S, et al. Spatial and temporal patterns of dengue incidence in northeastern Thailand 2006–2016. BMC Infectious Diseases. 2019;19(1):743. doi: 10.1186/s12879-019-4379-3.

23.	Srikiatkhachorn A, Rothman AL, Gibbons RV, Sittisombut N, Malasit P, Ennis FA, et al. Dengue--how best to classify it. Clinical infectious diseases : an official publication of the Infectious Diseases Society of America. 2011;53(6):563-7. Epub 2011/08/10. doi: 10.1093/cid/cir451. PubMed PMID: 21832264.

24.	Hause AM, Perez-Padilla J, Horiuchi K, Han GS, Hunsperger E, Aiwazian J, et al. Epidemiology of Dengue Among Children Aged < 18 Months-Puerto Rico, 1999-2011. The American journal of tropical medicine and hygiene. 2016;94(2):404-8. Epub 2015/12/30. doi: 10.4269/ajtmh.15-0382. PubMed PMID: 26711519; PubMed Central PMCID: PMCPMC4751950.

25.	Unnikrishnan R, Faizal BP, Vijayakumar P, Paul G, Sharma RN. Clinical and laboratory profile of dengue in the elderly. J Family Med Prim Care. 2015;4(3):369-72. Epub 2015/08/20. doi: 10.4103/2249-4863.161323. PubMed PMID: 26288776; PubMed Central PMCID: PMCPMC4535097.

26.	Secretaría de Salud M. LINEAMIENTOS PARA LA VIGILANCIA POR LABORATORIO DEL DENGUE Y OTRAS ARBOVIROSIS, INDRE (INSTITUTO DE DIAGNÓSTICO Y REFERENCIA EPIDEMIOLÓGICOS ‘DR. MANUEL MARTÍNEZ BÁEZ’). 2021. Available from: <https://www.gob.mx/cms/uploads/attachment/file/629265/Lineamientos_Dengue_Arb_V1-2021.pdf>.

27.	VanderWeele TJ. Mediation Analysis: A Practitioner's Guide. Annu Rev Public Health. 2016;37:17-32. Epub 2015/12/15. doi: 10.1146/annurev-publhealth-032315-021402. PubMed PMID: 26653405.

28.	MacKinnon DP, Dwyer JH. Estimating mediated effects in prevention studies. Evaluation review. 1993;17(2):144-58.

29.	Rijnhart JJM, Valente MJ, Smyth HL, MacKinnon DP. Statistical Mediation Analysis for Models with a Binary Mediator and a Binary Outcome: the Differences Between Causal and Traditional Mediation Analysis. Prevention Science. 2021. doi: 10.1007/s11121-021-01308-6.

30.	MacKinnon DP, Lockwood CM, Brown CH, Wang W, Hoffman JM. The intermediate endpoint effect in logistic and probit regression. Clinical Trials. 2007;4(5):499-513.

31.	Hayes AF, Preacher KJ. Statistical mediation analysis with a multicategorical independent variable. Br J Math Stat Psychol. 2014;67(3):451-70. Epub 2013/11/06. doi: 10.1111/bmsp.12028. PubMed PMID: 24188158.

32.	Alahmadi K, Al-Zahrani A. Spatial Autocorrelation of Cancer Incidence in Saudi Arabia. International journal of environmental research and public health. 2013;10:7207-28. doi: 10.3390/ijerph10127207.

33.	Anselin L. Local indicators of spatial association—LISA. Geographical analysis. 1995;27(2):93-115.

34.	Pro A. Optimized Outlier Analysis (Spatial Statistics) n.d [1/27/2022]. Available from: <https://pro.arcgis.com/en/pro-app/latest/tool-reference/spatial-statistics/optimizedoutlieranalysis.htm>.

35.	Thai KTD, Nishiura H, Hoang PL, Tran NTT, Phan GT, Le HQ, et al. Age-specificity of clinical dengue during primary and secondary infections. PLoS neglected tropical diseases. 2011;5(6):e1180-e. Epub 2011/06/21. doi: 10.1371/journal.pntd.0001180. PubMed PMID: 21713018.

36.	Guzman MG, Alvarez M, Halstead SB. Secondary infection as a risk factor for dengue hemorrhagic fever/dengue shock syndrome: an historical perspective and role of antibody-dependent enhancement of infection. Arch Virol. 2013;158(7):1445-59. Epub 2013/03/09. doi: 10.1007/s00705-013-1645-3. PubMed PMID: 23471635.

37.	Cisneros A, Díaz-Badillo Á, Cruz-Martínez G, Tovar R, Ramírez-Palacios LR, Jiménez-Rojas F, et al. Dengue 2 genotypes in the state of Oaxaca, Mexico. Archives of Virology. 2006;151(1):113-25.

38.	Annan E, Bukhari MH, Treviño J, Abad ZSH, Lubinda J, da Silva EAB, et al. The ecological determinants of severe dengue: A Bayesian inferential model. Ecological Informatics. 2023;74:101986. doi: <https://doi.org/10.1016/j.ecoinf.2023.101986>.

39.	Mweya CN, Kimera SI, Stanley G, Misinzo G, Mboera LE. Climate Change Influences Potential Distribution of Infected Aedes aegypti Co-Occurrence with Dengue Epidemics Risk Areas in Tanzania. PLoS One. 2016;11(9):e0162649. Epub 2016/09/30. doi: 10.1371/journal.pone.0162649. PubMed PMID: 27681327; PubMed Central PMCID: PMCPMC5040426.

40.	Centers for Disease Control and Prevention. Dengue: Clinal Presentation 2021 [7/26/2022]. Available from: <https://www.cdc.gov/dengue/healthcare-providers/clinical-presentation.html>.

41.	MacKinnon DP, Fairchild AJ, Fritz MS. Mediation analysis. Annu Rev Psychol. 2007;58:593-614. Epub 2006/09/14. doi: 10.1146/annurev.psych.58.110405.085542. PubMed PMID: 16968208; PubMed Central PMCID: PMCPMC2819368.
