# Supplementary material for: Direct and indirect effects of age on dengue severity: The mediating role of secondary infection
Source: PLoS Negl Trop Dis. 2023 Aug 9;17(8):e0011537. doi: 10.1371/journal.pntd.0011537 (PMC10441797; doi:10.1371/journal.pntd.0011537)
Supplement: Attachment — Submitted filename: Reviewers comments_responses PLoS NTD_5.25.23.docx [file pntd.0011537.s015.docx]

PNTD-D-22-01621

Dear *PLOS NTD editor* and peer reviewers,

We offer our sincere thanks for your thoughtful reviews of our manuscript, “Direct and indirect effects of age on dengue severity: the mediating role of secondary infection’. We made numerous revisions in accordance with your feedback. Your comments, our responses, and information about the changes are described in point-by-point detail below. We also uploaded a version of the manuscript in which all changes have been highlighted in yellow.

Comments and responses:

Reviewer 1

**Comment 1. The subject of this research is very interesting. However, I am skeptical about the results due to contradictory results in the analysis. For example, Table 4 reports a statistically significant negative total effect, and the total effect calculated manually using Equations 12-14 should have been consistent with the result generated by SAS in Table 4. However, this paper presents inconsistent or even completely opposite results.**

Response: Thank you for this comment. Based on this comment and comment 3, we used a generalized additive model with age as a continuous variable, and further conducted a mediation analysis using the CAUSALMED package in SAS. Please see highlighted tables, highlighted supplemental tables, and highlights within the manuscript that reflect these changes, which are consistent throughout the results section.

1. At The beginning of ***line 44, page 2*** the sentence reads ‘*We observed a nonlinear trend between age and severe dengue. There was a downward trend of severe dengue for individuals between 0 and 10 years and an upward trend above 10 years. The effect of age on dengue severity was no longer significant for individuals between 10 and 60 years after introducing infectivity status into the model. The mediating role of infectivity status in the causal model was 17%.*’
2. At The beginning of ***line 181, page 7,*** the sentence reads ‘*The mediation analysis was done using the SAS CAUSALMED procedure. The CAUSALMED procedure employs the counterfactual framework [31], where direct and indirect effects are defined in terms of counterfactual outcomes. The controlled direct effect (CDE) is the difference between the response and non-response outcome for a specific mediator value (3). The natural direct effect (NDE) is the difference between the response outcomes when the effect of the mediator is set to 0 (4), while the natural indirect effect (NIE) is the difference between the mediator levels when the response outcome is present (5) [32].*

*CDE = Y_1_m -Y_0_m ……………………………………………………………………………….………. (3)*

*NDE = Y_1_m_0_ -Y_0_m_0_ …………………………………………………………………………….………. (4)*

*NIE = Y_1_m_1_ -Y_1_m_0_ ……………………………………………………………………………………… (5)*

*Equation (6) shows the formula for the total effect (TE) decomposition and the percentage of the total effect that is mediated (PM), is therefore given calculated using the formula in equation (7) The definitions of these effects are applicable to both linear and nonlinear models since they are independent of the outcome and mediator [32].*

*TE = NDE + NIE ………………………………………….……………………………………………… (6)*

*PM = NIE/TE *100% ……………………………………………………..………………………….. (7)*’

3. At the beginning of ***line 268, page 11*** the sentence reads ‘*Table S3-S5 show the results from the GAM procedures. When controlled for other variables, males have 0.91 (CI: 0.86, 0.97) times the odds of females of having a secondary infection (Table S3). However, compared to females, males tend to have higher odds [1.40 (CI: 1.35, 1.46)] of severe infection after controlling for region and infectivity status (Table S5). While there exists higher odds of secondary infection in the Southeast [2.19 (CI: 1.76, 2.73)] and Northwest [3.10 (CI:2.30, 4.51)] regions compared to the Center region (Table S3), the odds of severe dengue is higher for the Center region compared to all regions, except for the Southeast region (Table S5). Figure 2 a and b show the GAM with severe diagnosis as the outcome variable. While Figure 2a models age on severe diagnosis, Figure 2b models age and infection status on dengue severity. Addition of infectivity status in model 2b resulted in a general non-significant pattern of association between individuals within the age ranges of 10 and 60 years and severe dengue. Both models, however, show a clear pattern of decreasing severity from 0 to about 10 years, and an increasing pattern above 60 years of age, although the confidence intervals also widened with increasing age. Figure 2c shows a steep increase in the association between age and infectivity status from 0 to 20 years, after which increasing age is associated with a gradual increase in secondary infection. Table 3 summarizes the results from the CAUSALMED procedure. The proportion of the NDE was 82.65%, indicating that the NIE or the percentage mediated was 17.35%.*

*Table 3: Results from mediation analysis*

|  | Estimate | Standard Error | LCI (Lower Confidence Limit) | UCI (Upper Confidence Limit) | P-Value |
| --- | --- | --- | --- | --- | --- |
| Total Effect | 0.0005 | 0.0001 | 0.0003 | 0.0007 | <.0001 |
| Controlled Direct Effect (CDE) | 0.0004 | 0.0001 | 0.0002 | 0.0006 | <.0001 |
| Natural Direct Effect (NDE) | 0.0004 | 0.0001 | 0.0002 | 0.0006 | <.0001 |
| Natural Indirect Effect (NIE) | 0.0001 | 0.0000 | 0.0001 | 0.0001 | <.0001 |
| Percentage Mediated | 17.3526 | 3.7902 | 9.9241 | 24.7812 | <.0001 |
| Percentage Due to Interaction | 0 | . | . | . | . |
| Percentage Eliminated | 17.3526 | 3.7902 | 9.9241 | 24.7812 | <.0001 |

4. At the beginning of ***line 297, page 13*** ‘*There is a nonlinear downward trend from 0-10 years and an upward trend between those greater than 10 years of age and dengue severity. However, the effect of age on severe dengue appears to be no longer significant for those 10 to 60 years after infectivity status was introduced into the causal model. The mediation analysis also revealed that infectivity status partially mediated the causal pathway between age and severe dengue by 17%.*’
5. At the beginning of ***line 302, page 13*** the sentence reads ‘*The steep decline of dengue fever severity from 0 to 10 years, and the steep increase for those 60 years and over, further suggests that infants and individuals 60 years and older will need close monitoring and possibly critical management of dengue infection.*’
6. At the beginning of ***line 336, page 14*** the sentence reads ‘*However, in our study, this was particularly true for individuals older than 60 years, and false for individuals between 0-10 years of age.’*

**Comment 2. The primary aim of this paper is to examine the mediating effect of secondary infection history on the association between age and dengue severity. However, the relevant results are limited, and most of the findings presented are not directly related to the research question, such as those in the Spatial Analysis section.**

Response: Thank you for this comment, we have added more details to the Results section to reflect the main aim of the manuscript. We have also modified the introduction section to clearly delineate the sub-aims of this paper. Beginning ***line 101, page 4,*** the paragraph reads ‘*This mediation analysis was performed to help provide information about the causal mechanism between age and dengue severity. We hypothesized that the effect of age on dengue severity would be insignificant after the introduction of infectivity status into a model with which age causes severe dengue, after controlling for confounding variables.* *We further characterized the spatial distribution of infectivity status for severe dengue across Mexico. While a secondary infection of dengue may be due to a homologous infection, a secondary infection that is associated with severe dengue is likely due to infection with a heterologous DENV serotype [21]. Hence, we expected to find spatial differences across infectivity status for those with severe dengue. Regional differences of infectivity may further be associated with a differential presence of DENV serotypes, which may potentially affect efforts to control dengue in areas with significant clusters of secondary infection*.’

**Comment 3. In this paper, infants under one year old are utilized as the control group. However, the number of individuals in other age groups is significantly higher than that of this control group, exceeding it by more than tenfold. This may seriously impact the statistical power of the analysis.**

Response: Thank you. This concern is addressed in response to comment 1. We kept age as a continuous variable in the model.

Reviewer 2 (Word Doc Review)

**Comment 1: Need to spell the first instance out**

Response: Thank you. This has been spelled out now beginning in ***line 36, page 2*** and the sentence reads ‘*Severe dengue occurrence has been attributed to increasing age and different dengue virus (DENV) serotypes that cause secondary infections and immune-enhancing phenomena.*’

**Comment 2: I don’t think you can control for this because it occurs after age, i.e., it is a post-intervention covariate.**

Response: We really appreciate that you pointed out this critical information. DENV serotype was removed from the model.

**Comment 3: Need to spell the first instance out.**

Response: Thank you. This was addressed in comment 1.

**Comment 4: The second sentence here seems to contradict the first. The first sentence says there is lifelong immunity against the infecting serotype; the second sentence says the infecting serotype can cause secondary infection. Please clarify.**

Response: We appreciate this observation. The second sentence has been removed to avoid miscommunication.

**Comment 5: I don’t see Figure 1 anymore.**

Response: Figure 1 has now been uploaded and you should be able to see it. Thank you for spotting this omission.

**Comment 6: Here and in other places where the word “prediction” is used- as stated in other places, the purpose of this analysis is not a prediction, it is to test causality. Suggest to change this to: “…with which age possibly causes dengue severity.”**

Response: Several modifications have been made throughout the manuscript. Beginning ***line 101, page 4*** the sentence now reads '*This mediation analysis was performed to help provide information about the causal mechanism between age and dengue severity. We hypothesized that the effect of age on dengue severity would be insignificant after the introduction of infectivity status into a model with which age causes severe dengue, after controlling for confounding variables.*'. Beginning ***line 153, page 6*** the sentence reads ' *The proportion of dengue severity across demographic variables and mediating were determined* ' and beginning ***line 155, page 6 the*** sentence reads *'The Type I error alpha value was set at 0.05 for all significance tests. Univariate analyses were conducted for descriptive purposes, and bivariate analyses were performed to determine associations between different independent variables and dengue severity.* '

**Comment 7: This is a bit confusing at this point- will you test how the effect of secondary infection is moderated by DENV serotype? Or are you testing how age’s indirect effect through secondary infection is moderated by DENV serotype? And for either, what would this add to the study’s purpose?**

**After reading through the manuscript, I would remove this because this was not actually presented.**

Response: Thank you. This sentence has been removed.

**Comment 8: Any exclusion criteria?**

Response: Yes. Beginning from ***line 116, page 4,*** this has been added to the manuscript and it reads '*Individuals with missing data on IgM, IgG laboratory results and/or dengue diagnosis were excluded from the analysis. The dataset had information about individuals meeting the inclusion criteria from 2012 to 2017.* '

**Comment 9: What is data entered as “other”?**

Response: Beginning ***line 133, page 5,*** the term 'other' has been defined and the sentences now read *'Disease classifications other than the DF categories were grouped as ‘other’ in the dataset. Data entered as ‘other’ or missing were excluded from the analysis.* '.

**Comment 10: Please check if this correction is accurate.**

Response: To improve clarity, the sentence has been modified to read '*Individuals with IgM positive, as well as IgG negative results were classified as having only a primary infection and no secondary infection'* beginning from ***line 141, page 5.***

**Comment 11: DENV should not be included as a confounder because it does not precede your exposure.**

Response: Information about DENV serotype has been removed from the covariates section as it was no longer controlled for in the model.

**Comment 12: Age should not be included as a covariate because it is already your exposure variable.**

Response: This is correct! Thank you for pointing out this error

**Comment 13: These were not defined in the covariates section. I am not sure they should be included because they do not precede age.**

Response: The final model used 'sex' and 'region' as covariates. Beginning ***line 199, page 8,*** the sentence now reads ' *For each model, sex and region were used as covariates.'.*

**Comment 14: It could be helpful if you did something similar with the direct effects. Or, if possible, put the direct, indirect, and total effects in a table, instead of Figure S7. Because there are several numbers, it’s hard to tell based on Figure S7 which numbers correspond to the direct, indirect, and total effects.**

Response: Because we use a GAM, Figure S7 has now been removed.

Once again, thank you for the helpful and constructive feedback. Thank you for giving us the opportunity to revise and resubmit our manuscript.

Sincerely,

The Authors
