# Supplementary material for: Direct and indirect effects of age on dengue severity: The mediating role of secondary infection
Source: PLoS Negl Trop Dis. 2023 Aug 9;17(8):e0011537. doi: 10.1371/journal.pntd.0011537 (PMC10441797; doi:10.1371/journal.pntd.0011537)
Supplement: Attachment — Submitted filename: Reviewers comments_responses PLoS NTD_7.10.23.docx [file pntd.0011537.s016.docx]

PNTD-D-22-01621

Dear *PLOS NTD editor* and peer reviewers,

We offer our sincere thanks for your thoughtful reviews of our manuscript, “Direct and indirect effects of age on dengue severity: the mediating role of secondary infection’. We made numerous revisions in accordance with your feedback. Your comments, our responses, and information about the changes are described in point-by-point detail below. We also uploaded a version of the manuscript in which all changes have been highlighted in yellow.

Comments and responses:

Use this section to provide overall comments, discuss strengths/weaknesses of the study, novelty, significance, general execution, and scholarship. You may also include additional comments for the author, including concerns about dual publication, research ethics, or publication ethics. If requesting major revision, please articulate the new experiments that are needed.

**Reviewer #1: It is advisable to consult with epidemiologists to review the analysis and the results of this study.**

Response: Thank you for this comment. This paper includes two Epidemiologists and one Biostatistician. Critical epidemiological tools used in this mediation analysis included the use of the directed acyclic graph (DAG) to inform our analysis, which was further improved based on feedback from previous rounds of review. We chose to do a mediation analysis to quantify how much of a direct and indirect effect age had on severe dengue and to determine how secondary infection status contributed to the causal pathway. We improved previous models, using a generalized additive model which better fits the relationship between age and severe dengue. This process was done iteratively and with constructive critique from the major review. Retention of age as a continuous variable in the generalized additive model, allowed us to perform further decomposition analysis to identify the percentage mediated using the ‘CAUSALMED’ package in SAS. However, the CAUSALMED package is limited in its ability to perform sensitivity analysis to test if the sequential ignorability assumption, which assumes that all confounders have been measured, has been violated. We have added this to the discussion section to explain this limitation. Beginning ***line 352, page 15***, the sentences read ‘*Lastly, the CAUSALMED package is limited in its ability to perform sensitivity analysis to determine if the sequential ignorability assumption made in the causal analysis [42] is robust. Although the assumption has been considered non-refutable because it cannot be directly tested from observed data, our exposure and mediating variables are unlikely to compromise the assumption as much as parameters that may involve non-randomization. For instance, people do not assign themselves an ‘age’ nor choose whether to be classified as having a primary or secondary infection.’.*

**Reviewer #2: Per a previous comment, I was still not able to see the figures, but assuming those are correct, all comments were addressed.**

Response: Thank you for all your feedback! We have double-checked and all the figures have been uploaded.

Once again, thank you for the helpful and constructive feedback. Thank you for giving us the opportunity to revise and resubmit our manuscript.

Sincerely,

The Authors
